# Brain changes: aerobic exercise for traumatic brain injury rehabilitation

**DOI:** 10.3389/fnhum.2023.1307507

**Published:** 2023-12-20

**Authors:** Taylor Snowden, Jamie Morrison, Meike Boerstra, Eric Eyolfson, Crystal Acosta, Erin Grafe, Hannah Reid, Justin Brand, Matthew Galati, Judith Gargaro, Brian R. Christie

**Affiliations:** ^1^Division of Medical Sciences, University of Victoria, Victoria, BC, Canada; ^2^Brain Changes Initiative, Concord, ON, Canada; ^3^KITE Research Institute, University Health Network, Toronto, ON, Canada; ^4^Island Medical Program and Department of Cellular and Physiological Sciences, The University of British Columbia, Victoria, BC, Canada

**Keywords:** traumatic brain injury, aerobic exercise, TBI intervention, aerobic intervention, concussion

## Abstract

**Introduction:**

Traumatic Brain Injury (TBI) accounts for millions of hospitalizations and deaths worldwide. Aerobic exercise is an easily implementable, non-pharmacological intervention to treat TBI, however, there are no clear guidelines for how to best implement aerobic exercise treatment for TBI survivors across age and injury severity.

**Methods:**

We conducted a PRISMA-ScR to examine research on exercise interventions following TBI in children, youth and adults, spanning mild to severe TBI. Three electronic databases (PubMed, PsycInfo, and Web of Science) were searched systematically by two authors, using keywords delineated from “Traumatic Brain Injury,” “Aerobic Exercise,” and “Intervention.”

**Results:**

Of the 415 papers originally identified from the search terms, 54 papers met the inclusion criteria and were included in this review. The papers were first grouped by participants’ injury severity, and subdivided based on age at intervention, and time since injury where appropriate.

**Discussion:**

Aerobic exercise is a promising intervention for adolescent and adult TBI survivors, regardless of injury severity. However, research examining the benefits of post-injury aerobic exercise for children and older adults is lacking.

## Graphical Abstract

A graphical representation of the study design and findings. 
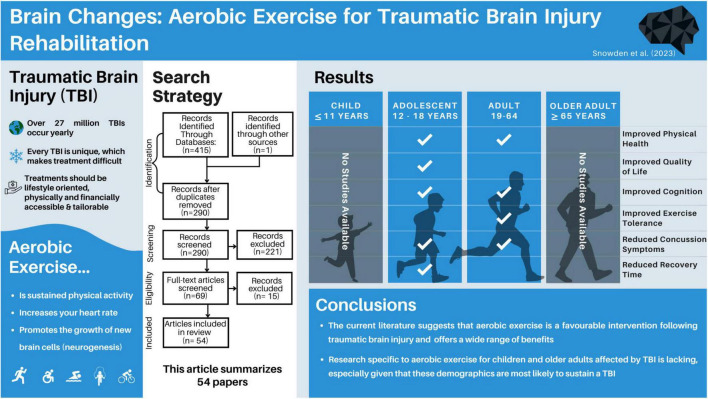

## 1 Introduction

Traumatic brain injuries are a global health issue, with more than 27 million treated injuries being reported yearly (James et al., 2019). To put this figure in context, the number of reported head injuries is greater than the entire population of some countries, like Australia (25.69 million) (Australian Bureau of Statistics, 2023). Despite this being a global health issue, treating individuals with traumatic brain injury (TBI) remains challenging. TBIs are often

called snowflake injuries due to their unique etiology, severity, affected population, and the burden they place on families and the health care system in any country. Given how unique each TBI can be, finding the best course of treatment for individuals remains challenging. Ideal treatments should be lifestyle oriented, physically and financially accessible, tailorable, and easily implementable. While aerobic exercise may meet many of these criteria, it is currently unknown how effective it is as a treatment for TBI across age and injury severity.

Aerobic exercise can be defined as low-to-vigorous-intensity, repetitive physical exercise performed for extended periods, that produce an elevation in heart rate. The term “aerobic” highlights how the body uses oxygen to meet energy requirements through aerobic metabolism (McArdle et al., 2006; Plowman and Smith, 2007). Both subjective measures (relative perceived exertion, talk tests) and objective measures (heart rates, oxygen intake/output) are common ways of assessing an aerobic exercise prescription, with the gold standard being a maximal oxygen consumption test (Lee and Zhang, 2021). The current Government of Canada recommends that healthy adults aged 18–64 get at least 2.5 h per week of physical activity, focusing on aerobic activity in 10 + min sessions (Government of Canada, 2018). While these guidelines are helpful for general fitness, very few standardized recommendations exist for aerobic exercise following TBI, and most of the graded recommendations are based on mild TBI (Parachute, 2017).

It is worth exploring aerobic exercise as a rehabilitation method for brain injury for a number of reasons. First, it promotes physical health, and cardiovascular fitness. Additionally, it has been shown to promote cognitive and mental health (Ruegsegger and Booth, 2018), both of which can be impacted by TBI (Langlois et al., 2006; Haarbauer-Krupa et al., 2021). Aerobic exercise is a potentiator of neuroplasticity, the brain’s ability to rewire, reorganize and form new neural connections. In animal models, it has been extensively studied as a means to stimulate neurogenesis, the brain’s ability to form and integrate new neurons (van Praag et al., 1999; Farmer et al., 2004; Nokia et al., 2016). It has been shown to improve cognitive functions commonly impacted by TBI, including improving attention, memory and processing speed in children and adults (Khan and Hillman, 2014; Young et al., 2015), as well as reducing levels of depression and anxiety (Carek et al., 2011). Given that TBI-survivors have a higher likelihood of poverty (Young and Hughes, 2020) and disability (Hyder et al., 2007), the low cost and adaptable nature of aerobic exercise makes it an appealing therapeutic option.

An important consideration for brain injury interventions is that TBI outcomes can differ depending on the age at which the injury was sustained. Children are more likely to show initial improvements, only to later have disrupted brain development leading to cognitive and behavioral issues that manifest over time, potentially impacting academic performance and social relationships (Anderson et al., 2009). Adolescent children aged 12 to 17 may have a more complicated TBI experience, as this is a period of complex cognitive, hormonal and physical growth. For example, studies focused on TBI in adolescents have observed significant risks of further mental and physical health challenges (Ilie et al., 2014) and impaired socially adapted decision capacity, compared to non-brain-injured peers (Beauchamp et al., 2019). For adults, especially as they reach middle-to-older age, post-TBI challenges can span from difficulties with the return to work, early cognitive decline, and slowed recovery timelines (Rabinowitz et al., 2021). Despite these complexities, developing brains tend to have greater neuroplastic potential, and may be more receptive to post-injury intervention. Given these age-associated TBI outcomes and risks, it is essential to consider the affected individual’s age, as the injury’s implications and recovery trajectories differ across age groups.

Previous systematic review papers on the effects of aerobic exercise following traumatic brain injury have been limited by focusing on the severity of injury [i.e., concussion (Howell et al., 2019; Langevin et al., 2020)] or specific outcome measures [i.e., quality of life (O’Carroll et al., 2020) and cognition (McDonnell et al., 2011)]. To better understand how aerobic exercise may be used as a treatment following TBI, this systematic scoping review aims to encapsulate the literature examining aerobic exercise-based interventions following TBI, separated by injury severity and age. Given the diversities of the injury and the intervention, a scoping review was deemed an appropriate methodology to synthesize the vast body of evidence in this field. The aim is to present a comprehensive review of the literature on aerobic exercise as a post-TBI intervention, in order to better determine what interventions are supported and which areas require more investigation.

## 2 Methods

### 2.1 Registration

This scoping review was conducted in accordance with the Preferred Reporting for Systematic Reviews and Meta-analyses extension for scoping reviews (PRISMA-ScR) (Tricco et al., 2018) and was guided using pre-determined frameworks for scoping reviews (Arksey and O’Malley, 2005; Levac et al., 2010; Tricco et al., 2018). An overview of this framework includes: identifying the research question, relevant studies, study selection, data extraction/data charting, and summarizing and presenting the results. This review’s objectives, eligibility criteria, preliminary study characteristics, and indicator papers were determined before starting the study. In accordance with PRISMA-ScR, the protocol was not registered; however, is available by request.

### 2.2 Research question

What is the evidence surrounding aerobic exercise to improve cognitive, mental and physical health following all severities of traumatic brain injury in children, adolescents and adults?

### 2.3 Eligibility criteria

Inclusion and exclusion criteria were determined *a priori* and selected to capture focused results related to the research question (Supplementary Table 1).

### 2.4 Information sources

Three databases (i.e., Web of Science, PubMed, PsychInfo) were selected as digital search engines for this review paper. These databases were selected based on their missions to deliver quality clinical and medical materials. The first search occurred on May 01, 2023, and a final search was conducted on August 01, 2023. Two authors (TS and JM) conducted these searches individually and compared search results to ensure the reliability of the search terms.

### 2.5 Search

To identify eligible articles, four search term blocks were used (Supplementary Table 2). Four search blocks were used, each to assist in finding related articles: Traumatic Brain Injury, Aerobic Exercise, Intervention, Review. The first three blocks used AND modifiers between, while the fourth block used a NOT modifier to reduce the number of review papers included in the search results.

### 2.6 Selection of sources of evidence

**FIGURE 1:**
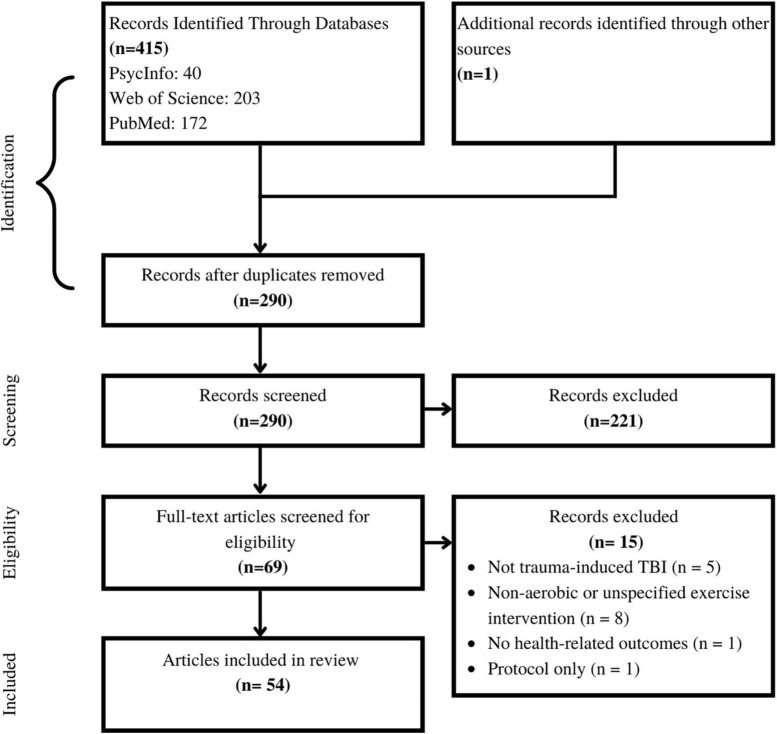
Selection of sources of evidence. The authors started with 415 papers. Following systematic duplication removal, screening, and eligibility assessment 54 papers were included in this review.

The first and second authors individually exported the results into Zotero citations manager for screening and selection. To start, duplicate titles were removed, then titles and abstracts were screened for initial eligibility. The remaining papers were read in entirety, and full eligibility was assessed, as determined by the *a priori* inclusion criteria. The first and second author compared their final papers for inclusion, and any discrepancies were resolved by consensus. The number of papers at each step is available in Figure 1.

### 2.7 Data charting

Data charting was split between team members (TS, JM, MB, EE, CA, EG, HR, JB), and overseen and confirmed by TS and JM. Google Sheets was used to chart data, and specific variables of interest were identified before data charting. In addition to the standard variables (e.g., type of study, participant demographics, time since injury, intervention details), a notes and considerations section was added such that the charter was able to make note of anything pertinent in the study that was not immediately captured in the primary data charts. The results tables are simplified versions of the complete data charting used in this review.

### 2.8 Data items

The specific data items and rationale used in the data charting can be found in Supplementary Table 3.

### 2.9 Synthesis of results

Following the data charting, TS reviewed all charting and papers to ensure their alignment with the goals of the paper. While data charters made notes about considerations for each study, the authors did not conduct any formal critical appraisals or bias scoring in alignment with PRISMA-ScR protocols. However, should this scoping review inspire systematic reviews, the authors encourage including bias scoring or other critical metrics of the included data. Upon completion of data charting, papers were grouped based on participant age and severity of TBI, and a narrative summary was composed.

## 3 Results

### 3.1 Selection of sources as evidence

The first and second authors individually searched Web of Science, PubMed and PsychInfo databases, and found a total of 415 articles. After removing duplicate items, 290 articles were screened. After screening the title and abstract of each paper, 69 papers remained for full-text reading. Of the 69 papers, five were excluded due to wrong injury type, eight were excluded for not including an aerobic intervention, one was excluded as it did not contain primary research data, and one was excluded for not including any health-related outcome measures, leaving 54 papers in this review (Figure 1).

### 3.2 Characteristics of sources of evidence

Of the included articles, three overarching research designs emerged, including case series, randomized controlled trials, and non-randomized pre-post intervention studies. All citations were presented as primary, peer-reviewed articles, as defined in the inclusion and exclusion criteria. In studies encompassing multiple pre-defined age groups, the results were reported separately within each respective category if separate age-based analyses were conducted. However, if the age groups were combined, the study findings would be reported based on the category that corresponds to the mean age of the participants. Therefore, while thirty-four of the studies included adults (aged 18 +), 23 included adolescents (aged 12–17, inclusive), and five included children under 12, the main grouping of TBI severity could only be sub-sectioned into adults (*n* = 32) and adolescents (*n* = 22), given that all studies reported a mean age above 12.

### 3.3 Results of individuals sources of evidence

**FIGURE 2:**
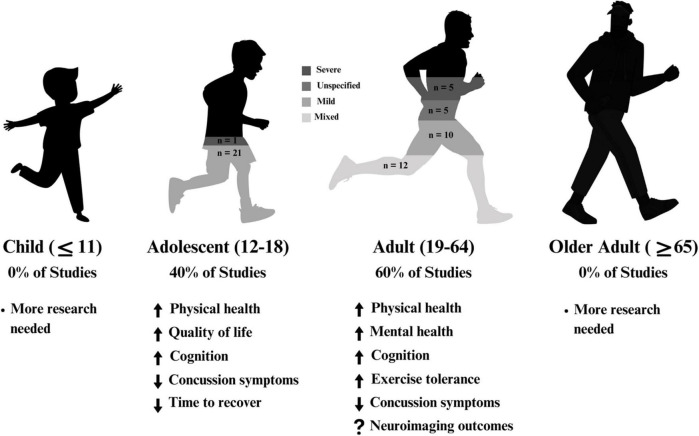
A summary of results across age within the included articles. A majority of studies focussed on adults (aged 19–64), and the remaining studies focussed on adolescents (aged 12–18). 57% of studies solely examined mild traumatic brain injury.

The following sections are divided by reported severity of injury (severe, mixed, mild, unspecified) and further subdivided by reported age group (adult, adolescent). Studies with similar outcome measures (mental health, physical health, cognitive health) are presented within these subcategories. Figure 2 provides an overview of the selected included articles.

### 3.4 Severe brain injury

Six of the included studies exclusively studied individuals with severe brain injuries. Five papers examined adults (Hassett et al., 2009; Corral et al., 2014; Chanpimol et al., 2017; Curcio et al., 2020; Wender et al., 2021), while one presented a case report on an adolescent female (Tiwari et al., 2018). Full results are presented in Table 1.

**TABLE 1:** Summary of the effects of aerobic exercise intervention following severe traumatic brain injury.

Refe-rences	Study Design	Aim/ objective	Participant details	Time since injury	Intervention timeline	Intervention details	Interven-tion location	Aerobic modality	Participant adherence	General outcome measures	Specific outcome measures	Results related to aerobic exercise
Chanpimol et al., 2017	Case series	To evaluate the effects of a Kinect-based virtual reality (VR) intervention using commercially available motion capture games on balance outcomes for an individual with chronic TBI and to assess the feasibility of this intervention for eliciting cardiovascular adaptations	*n* = 1; adult aged 37; male	11 years	2x sessions for 50–60 min per week for 8 weeks	VR training would consist of mini games which would adress domains of dynamic balance, static balance, and cardiovascular fitness that were appropriate for the participant based on clinical judgment. Rest breaks were allowed as required.	Clinic-based	Mini supervised VR games to challenge cardiovascular systems	The single participant completed the intervention	Balance; cardiovascular health	Dynamic Gait Index (DGI), Functional Reach Test (FRT), Limits of stability (LOS) test, resting HR (RHR); HR at end (HRe)	Significant decline in HR at end and time in training range with an increase in total activity time; improved dynamic balance
Corral et al., 2014	Pre-post intervention study	To determine if exercise in people with TBI can increase circulating progenitor cells and if there are any accompanying physical or psychological benefits of such intervention.	Exercise electro-stimulation group: *n* = 5; cycling program group: *n* = 5; adults aged 35 ± 7; males	At least 1 year	Exercise electro-stimulation group: 3 sessions per week for approximately 8 weeks; cycling program: 3 days per week for 12 weeks	Exercise electro-stimulation group intervention was not defined. Cycling program: warm up with 5 min of abdominal work and cycling until reaching 60% of maximum workload, followed by 3 × 12 min of interval work at 60–80% max workload, 3 min of active recovery at 60%, ending with gently cycling for 5 min and stretching.	Exercise electro-stimulation group: University Exercise Physiology Unit (3 sessions/week) and at home (2 sessions/week); cycling group: not specified	Exercise electro-stimulation group: not specified, but participants engaged in endurance, resistance, and proprioceptive exercises; cycling program: cycling, with a focus on endurance	2/5 participants completed the exercise electro-stimulation group; 5/5 participants completed the cycling group; 5/6 participants completed the intermittent hypobaric-hypoxia and muscle electro-stimulation group; 4/5 participants completed the control group	Aerobic capacity; circulating progenitor cell levels; physical stress; psychological stress	VO2 uptake; CDC34 + quantification in peripheral blood by staining and flow cytometry assay; Verbal Memory-RAVLT; Trail Making Test (TMT A and B); Stroop Test; Wechsler Adult Intelligence Scale (WAIS III); Tower of London tests; Reduced Paced Auditory Serial Addition Test (PASAT-G);	Exercise electro-stimulation group: improved aerobic capacity and increased circulating progenitor cell levels in peripheral blood in the last 3 weeks of intervention; cycling group: PASAT-G test improvement and significant increase in VO2 uptake
Curcio et al., 2020	RCT	To examine the effects of aquatic training on balance, gait, activities of daily living, and quality of life in severe TBI survivors	Aquatic intervention: *n* = 10; adults aged 37.4 ± 15.3; males and females; land control: *n* = 10; adults aged 43.0 ± 14.1; males and females	Acquatic interven-tion: 5.8 ± 2.6 months; land control: 4.8 ± 2.7 months	3 × 45 min sessions per week for 4 weeks	In the pool, participants did a warm up for 5 min consisting of breathing exercises and arm movements, followed by 20 min of a repetitive exercise sequence, and 20 min of step exercises.	Rehabilitation hospital	Acquatic therapy	22 participants initially; 2 dropped out (1 from intervention group and 1 from control group) before assessment	Balance (main outcome measure); disability; gait; quality of life; spasticity	Berg balance scale (BBS); modified Barthel index (MBI); Disability Rating Scale (DRS); Tinetti Gait Balance Scale (TBG); Quality of Life After Brain Injury (QOLIBRI); Modified Ashworth Scale (MAS)	Significantly increased scores for BBS, MBI, BBG, and QOLIBRI after intervention compared to baseline
Hassett et al., 2009	RCT	To compare the effects of supervised fitness center-based exercise with unsupervised home-based exercise on cardiorespiratory fitness and psychosocial functioning in people with TBI	Fitness center group: *n* = 32; adults aged 35.4 ± 14.6; home-based group: *n* = 30; adults aged 33 ± 11.8	Not reported; but participants recruited from inpatient admissions	3 × 1 h sessions per week for 12 weeks	5 min warm up; 20 min strength training for 6 muscle groups (quadriceps, plantar flexors, abdominals, pectorals, triceps, back extensors) 2 sets of 15 reps or 3 sets of 10 reps (total 180 reps); 30 min continuous cardiorespiratory fitness training that was symptom limited and moderate intensity (breathing hard but able to talk); 5 min cool down; walking/jogging exercise for at least 1 fitness session per week.	Fitness center and home-based	Walking or jogging	Adherence defined as ≥36 sessions over 12 weeks; better adherence in fitness center group (72 SD 25) vs. home-based 44 SD 42 (higher percentage of completed sessions)	Cardiorespira-tory fitness; psychosocial functioning	Primary physical: maximal velocity; distance; peak HR; % HR max; secondary physical: BMI; waist to hip ratio (WHR); waist circumference; goal attainment: #goals set; %goals achieved; psychological: depression; anxiety; stress; vigor; tension-anxiety; depression-dejection; anger-hostility; fatigue; confusion-bewilderment. Outcomes measured at baseline; at completion of intervention; and 3 months-post intervention using modified 20-m shuttle test (walk/jog along 20-m track with increasing speed).	Both groups improved in fitness as measured by distance and velocity; no differences between fitness center and home-based groups; no differences in psychosocial functioning; both interventions equally effective at improving cardiorespiratory fitness
Tiwari et al., 2018	Case series	To examine the effects of home based circuit training on gait, energy expenditure, and functional performance following TBI	*n* = 1; adolescent aged 17; female	2 years	35 min sessions 4x per week for 4 weeks	5 min warm up; 30 min circuit training; 10 exercises, 10 reps each in 1 circuit. Repeat in same order for 30 min; number of circuits performed progressed over the 4 weeks; Exercises (in order performed): lateral walking with elastic resistance bands, lunges, lateral lunge, sideways walking with squats, sit to stand transitions from a chair, squats, tall kneeling with arm raises, tall kneeling proprioceptive neuromuscular facilitation D-2 pattern, marching, hamstring and calf stretches	Home-based	Circuit training	100%	Balance; mobility; strength	6-Min Walk Test (6MWT); physiological cost index (PCI = HR walking-HR resting/walking speed); gait speed (meters/second); Canadian Occupational Performance Measure (COPM) performance score and satisfaction score	Improved distance in 6MWT; increased PCI score; decreased resting HR; improved gait speed; improved COPM; improved COPM satisfaction suggesting improved self-perception of occupational performance
Wender et al., 2021	RCT	To determine the feasibility of an aerobic exercise intervention for severe TBI survivors, and assess how aerobic exercise impacts neuropsychological function and brain structure	Exercise: *n* = 2; active control: *n* = 3; adults aged 27–49; males and females	1–27 years	3 × 30 min sessions for 12 weeks	TBI participants were randomly assigned to an aerobic exercise (recumbent cycling) or active control (stretching) group. The aerobic exercise group maintained at least 60% of their maximal HR during the sessions.	Clinic-based	Recumbent cycling	100%	Cardiorespi-ratory fitness; magnetic resonance Imaging; neuropsy-chological testing	Rey Auditory Verbal Learning Test; Symbol Digit Modalities Test; hippocampal and thalamic brain volume; peak oxygen consumption using the incremental exercise test	No between group differences on any measure; large effects of exercise were observed on the RAVLT and SDMT; exercise condition was associated with larger increases in left hippocampal and right thalamus volumes

#### 3.4.1 Adults

All studies included at least two time points (pre- and post-interventions). In most cases, individuals were adults when they sustained their TBI, with time since injury ranging across studies from unspecified inpatient admissions (Hassett et al., 2009) to 27 years post injury (Wender et al., 2021), however, most interventions occurred within 1–2 years post-injury. Intervention timelines ranged from 4-weeks to 12-weeks, typically 3 times per week for 30–60 min. Engagement in aerobic activity varied between studies; two used recumbent cycling for their aerobic component (Corral et al., 2014; Wender et al., 2021), one used a virtual reality platform (Chanpimol et al., 2017), one used aquatic therapy (Curcio et al., 2020), and the last used walking and jogging (Hassett et al., 2009). All studies were conducted in a clinic or otherwise supervised setting, and Hassett et al. (2009) also included a home-based exercise group.

Outcomes of interest across these studies included fitness, balance, quality of life, mental health, as well as brain structure and functions. Hassett et al. (2009), Corral et al. (2014), and Chanpimol et al. (2017) all observed improvements in measures of cardiovascular fitness across their interventions. Curcio et al. (2020) observed improvements in multiple measures of balance, and quality of life, while Wender et al. (2021) found large effects of exercise on measures of verbal memory and processing speed as compared to a stretching/toning control group. Additionally, Wender et al. (2021) reported greater increases in left hippocampal and right thalamic volumes, however this study included 2–3 individuals per group.

Some considerations of these studies include the limited definitions of aerobic or cardiovascular activity; only two studies included target heart rate or effort zones (Corral et al., 2014; Wender et al., 2021). Only one study included a home-based intervention and found decreased adherence in that group compared to clinic-based interventions. It is also important to consider the sample sizes in terms of adequate power and statistical analyses.

#### 3.4.2 Adolescents

One case report examined the effects of aerobic exercise on a 17-year-old female who sustained their TBI in adolescence (2 years post-injury) (Tiwari et al., 2018). The home-based intervention included 4 × 35-min weekly sessions, for 4 weeks. This circuit-style intervention included a mixture of resistance exercises and cardiovascular training. Similar to adults with severe TBIs, researchers found improved cardiovascular health, quality of life, and increased gait speed.

### 3.5 Mixed injury severity

Twelve studies included mixed severities of mild, moderate and/or severe in their aerobic intervention studies (Bhambhani et al., 2005; Brown et al., 2005; Schwandt et al., 2012; Wise et al., 2012; Bellon et al., 2015; Chin et al., 2015, 2019; Weinstein et al., 2017; Morris et al., 2018; Ding et al., 2021; Romanov et al., 2021; Tomoto et al., 2022), all of which reported on adults aged 18–65. Full results can be found in Table 2.

**TABLE 2:** Summary of the effects of aerobic exercise intervention following mixed-severity traumatic brain injury.

Refe-rences	Study design	Aim/ objective	Participant details	TBI demo-graphics	Time since injury	Interven-tion timeline	Intervention details	Interven-tion location	Aerobic modality	Participant adherence	General outcome measures	Specific outcome measures	Results related to aerobic exercise
Bellon et al., 2015	RCT	To determine the efficacy of a 12-week walking program for TBI survivors	*n* = 69; adults aged 43.7 ± 15.8; males and females	Mild *n* = 10; moderate *n* = 10; severe *n* = 35; unknown *n* = 13	Mean 100.5 ± 119.9 months	Daily walking for 12 weeks	Participants were given pedometers to count steps. Participants completed a 1-week baseline to establish a baseline step count. Participants were encouraged to increase their weekly steps by 5% each week, until they reached a 40% increase at week 8, and maintained that level for the remaining 4 weeks.	Home-based	Walking	Not reported, but of 123 enrolled participants, 69 completed all three time-points (baseline, 12-, 24-weeks).	Perceived depression; perceived stress	Perceived Stress Scale; Centre for Epidemiological Studies-Depression	Increased walking over 12-weeks; reduced depressive symptoms; reduced perceived stress
Bhambhani et al., 2005	Pre-post interven-tion study	To examine time course of body composition and cardiovascular changes following routine TBI rehabilitation and circuit training	*n* = 14; adults aged 18–52; males and females	Moderate and severe TBI	17.2 ± 17 months	3x circuit training sessions per week for 12 weeks	Each session included 5–10 min warm up, followed by 45 min of circuit training (15–20 min of cycling, arm cranking or treadmill walking, 15–20 min of resistance training, 5 min cool down). Participants aimed to keep their HR at 60% of their HRR.	Clinic-based	Recumbent cycling, arm cranking, treadmill walking	Not reported, but of 26 enrolled participants, 14 completed the circuit program and 5 test trials.	Body composition; peak cardiorespira-tory response	Body Mass Index;% Body Fat; Basal Metabolic Rate; Echocardiogram	No significant changes on body composition; improved cardiorespira-tory response (peak power, oxygen uptake and ventilation rate)
Brown et al., 2005	RCT	To compare body weight support treadmill training to conventional over ground gait training	*n* = 20; adults aged 20–57; males and females	Moderate and severe TBI	7–23 years	2x body weight support treadmill training for 15 min per week for 14 weeks	Starting speed was the fastest participants could handle and was increased as tolerated over the course of 3 months. If a rest was needed, the clock was stopped and continued when participants resumed.	Residential rehabilitation center	Body weight supported treadmill training	Not reported	Walking and stepping ability	Functional Ambulation Category; functional reach; Timed Up and Go; gait velocity; step width; step length differential using instrumented gait mat	Increased walking for both training; significant improvement in step length differential for conventional over ground gait training
Chin et al., 2015	Pre-post interven-tion study	To examine cognitive function in individuals with TBI prior to and after participation in an aerobic exercise training program	*n* = 7; adults aged 33.3 ± 7.9; males and females	Chronic, non-penetrating; mild *n* = 4; moderate *n* = 3; *n* = 5 injuries due to falls	4.0 ± 5.5 years	3 × 30 min per session of a vigorous intensity exercise on treadmill per week for 12 weeks	Participants completed neuropsychological assessments and self-report questionnaires prior to performing a treadmill cardiopulmonary exercise test to volitional exhaustion. The target HR for exercise training was 70–80% of the participants’ HRR and was calculated as based on the HR response from the baseline cardiopulmonary exercise test. Speed and/or grade were adjusted as needed to maintain the exercising HR within the target range. An additional 5–10 min of warm-up and cool-down were also performed within each training session.	Clinic-based	Vigorous-intensity exercise on treadmill	Not reported	Cardiorespira-tory fitness; cognitive function; depression; sleep quality	Trail Making Test Part A (TMT-A); Trail Making Test Part B (TMT-B); Repeatable Battery for the Assessment of Neuropsycho-logical Status (RBANS); Pittsburgh Sleep Quality Index (PSQI) and Beck Depression Inventory; version 2 (BDI-II); oxygen consumption rate; work rate	Improvements in cognitive function were observed with greater scores on the TMT-A, TMT-B, and RBANS total scale; no changes in measures of the PSQI and BDI-II which tested sleep quality and depression; magnitude of cognitive improvements was strongly related to the gains in cardiorespira-tory fitness
Chin et al., 2019	Pre-post interven-tion study	To examine the effect of aerobic exercise training on oxygen uptake on-kinetics during treadmill walking in individuals with TBI	*n* = 7; adults aged 33.3 ± 7.9; males and females	Chronic, non-penetrating; mild *n* = 4; moderate *n* = 3; *n* = 5 injuries due to falls	4.0 ± 5.5 years	3 × 30 min per session of a vigorous intensity exercise on treadmill per week for 12 weeks	Participants were initially evaluated for exercise test by performing two square-wave bouts of exercise at moderate intensity that was separated by 8 min of active recovery. The target HR for exercise training was 70–80% of the participants’ HRR and was calculated as based on the HR response from the baseline cardiopulmonary exercise test. Speed and/or grade were adjusted as needed to maintain the exercising HR within the target range. An additional 5–10 min of warm-up and cool-down were also performed within each training session.	Clinic-based	Vigorous-intensity exercise on treadmill	Not reported	Oxygen uptake on-kinetics; performance fatigability	HR; speed/grade on the treadmill	Faster oxygen uptake on-kinetics observed for both the absolute and relative intensity; suggesting improved performance in individuals with TBI
Ding et al., 2021	RCT	To investigate the feasibility of a community-based aerobic exercise intervention for people with TBI	*n* = 9; adults aged 18–65; males and females	Diagnosed mild to severe TBI; mild *n* = 6, moderate to severe *n* = 3	20 ± 22 months	3 months of aerobic exercise training; 20 min × 3 days for first week, increase to 30 min × 5 days or 50 min × 3 days by end of fourth week and for the remaining 8 weeks	Participants wore HR monitors. A total of 5 min warm up and cool down before and after each aerobic training session. Exercise intensity gradually increased training from 50–60% of max HR to 70–80% of max HR. First aerobic training session was conducted with a certified trainer, the rest from home or a local fitness center.	Home-based or community fitness center	Not specified; participants chose any modality preferred	Participant compliance (ratio of completed to prescribed exercise sessions that participants achieved their target HR) in aerobic exercise training (AET) group was 40% to 91% (media*n* = 76%). Attrition: initially 10 participants in the AET group, but 1 withdrew because of fatigue and muscle pain.	Cardiorespira-tory fitness; cognitive function; health status	Peak oxygen uptake (VO2peak); Flanker Inhibitory Control and Attention Test; List Sorting Working Memory Test; Picture Sequence Memory Test; Dimensional Change Card Sort Test; Pattern Comparison Processing Speed Test; Picture Vocabulary Test; Oral Reading Recognition; Patient-Reported Outcomes Measurement Information System	AET group had a trend of improved VO2peak (8%) compared to stretching and toning group (−4%); 7 of 9 AET participants had improved VO2peak (6% to 28%) after the 3 month intervention; no significant improvement in cognitive assessment performance; small, but significant improvement in depression, anxiety, and anger
Morris et al., 2018	Case series	To examine the feasibility of introducing aerobic physical exercise programs to those with severe TBI, which includes computerized cognitive training	*n* = 5; adults aged 19–56; males	Moderate or severe TBI; 3–8 or 9–13 on the GCS; no previous history of moderate or severe TBI; no aphasia, amnesia had resolved	24–91 days	8 weeks	Aerobic exercise occurred 3x per week for 8 weeks at 50–70% HRR for 45–60 min, including a 10 min warn up and cool down.	Hospital	Active/ passive exercise trainer or recumbent cycling, dependent on patient ability (changed over the course of the study based on recommenda-tion by physical therapist)	2 of 5 participants were only able to complete about half of the sessions, but percent adherence was calculated to be 87–100% in any given patient.	Feasibility outcomes; neuropsy-chological testing (mentioned in methods but not results)	Number of adverse events reported; adherence to the aerobic exercise program (session durations and number of session attended)	Weak correlation between ratings of perceived effort and HRR; no serious adverse events occurred in participants undergoing intervention
Romanov et al., 2021	RCT	To compare how an adapted physical exercise program including endurance, strength, balance, stretching, and cognitive exercises influences muscular ability, attention process and general workability in TBI patients	*n* = 25; adults aged 41.1 ± 9.7; males	Moderate to severe TBI	2 years	8 weeks	Regular Rehab program: 5 days a week (Monday to Friday), morning exercise (45 min each day), and brain gymnastics (45 min, only Tuesday and Thursday). Morning exercise included: endurance exercises to improve circulation, strength exercises to empower body, and stretching exercises to improve flexibility. Adapted Physical Exercise: 90 min, 2x per week (in addition to regular rehab program), 30–40 min, nordic walking with gymnastic rod.	Center for people with acquired brain injury	Nordic walking with gymnastic rods	100%	Attention process; motor and functional ability	Chair Stand Test; Bicep Curl Test; Chair Sit and Reach Test; 6-min Walk Test; Berg’s Balance Scale; Standardized d2 Test (tests attention process)	Both groups showed improvements across all tasks; experimental group showed greater improvements than Regular Rehab Program but differences were not significant; experimental group showed statistically significant improvement on Standardized d2 Test, indicating improved attention compared to other group
Schwandt et al., 2012	Pre-post interven-tion study	To determine the effectiveness of an aerobic exercise program on depression symptoms following TBI	*n* = 4; adults aged 19–48; males and females	Moderate to severe TBI	11 months–7.2 years; mean 2.6 years	3x per week for 12 weeks	Each session included a: 10 min warm up, 30 min at predetermined power output, and 10 min cool down. Participant predetermined power output was 208–(0.7 × age). Participants raised their HR above 70% of age-predicted maximum HR, and had a perceived exertion of 5 or 6 on Borg Scale, with systolic blood presure not >220 mm Hg. Training intensity was maintained 5–10 W below peak workload achieved on baseline testing.	Physio gym at rehabilitation hospital	Cycle ergometry, treadmill, or recumbent step machine; choice made by participant and researcher based on physical limitations, safety, and ability to reach certain thresholds	76–81%, mea*n* = 78%	Aerobic capacity; depressive symptoms; program perception questionnaire; self-esteem	Hamilton Rating Scale for Depression; Rosenberg Self-Esteem Scale; Borg Scale of perceived exertion; Peak power output; HR at fixed power output	Increased peak power output; decreased HR; decreased perceived exertion on Borg Scale; lower scores on Hamilton Rating Scale for Depression (i.e., less depressed); improved Rosenberg Self Esteem Scale scores (i.e., increased self-esteem)
Tomoto et al., 2022	Pre-post interven-tion study	To determine if aerobic exercise improves carotid arterial compliance in adults with chronic TBI	TBI: *n* = 19; control: *n* = 19; adults aged 26–61; males and females	Mild, moderate, and severe TBI; mild *n* = 11; moderate to severe *n* = 8	6 months–6 years	3 × 20 min sessions for the first week, followed by 3 × 50 min Or 5 × 30 min sessions for the remaining 11 weeks	TBI participants were split into an Aerobic Exercise Training (AET) group, and a Stretching and Toning (SAT) group. The intensity, frequency and duration of the AET program was based on an individuals maximal HR, and progressively increased as participants adapted to the workload. Week 1: 50–60% maximal HR; Week 2–12: 70–80% maximal HR. Participants started with a 5 min warm up, followed by the aerobic component, and then a 5 min cool down.	One session in clinic, remaining sessions in the community	Any mode of aerobic activity, as long as the HR goals were maintained	3 of 19 participants did not complete the intervention (1 lost to follow-up, 1 had surgery, 1 had muscle pain); compliance in the AET group ranged from 40–91%	Carotid arterial compliance (CAC); cerebral blood flow (CBF); cerebrovas-cular resistance (CVR)	CAC: tonometry and ultrasonography at the common carotid artery; CBF: ultrasonography at the bilateral internal carotid and vertebral arteries; pulsatile CBF: transcranial Doppler ultrasonography at the middle cerebral arteries; CVR: calculated as mean arterial pressure divided by total CBF	Increased CAC; improved VO2 max and decreased systemic blood pressure was observed following AET compared to SAT, but not statistically significant; increases in CAC were associated with decreased pulsatile CBF
Weinstein et al., 2017	Pre-post interven-tion study	To examine if 12-weeks of aerobic exercise changes mood in adults with chronic TBI symptoms	*n* = 10; adults aged 32.9 ± 6.5; males and females	Mild, moderate, and severe TBI; mild *n* = 11; moderate to severe *n* = 8	At least 6 months; mean 6.6 years	3 × 30 min sessions for 12 weeks	Participants were allowed 5–10 min of warm up and cool down. Participants aimed to have their HR between 70 and 80% of their maximum. Aerobic walking was performed supervised on a treadmill.	In clinic	Treadmill walking	12 participants were enrolled, 10 participants completed the program	Mood	Profile of Mood States Short Form	Less mood disturbances at week 12 compared to baseline
Wise et al., 2012	Pre-post interven-tion study	To determine the effect of exercise on exercise maintenance, depression, quality of life and mental health in adults with TBI and depression	*n* = 40; adults aged 18–55; males and females	Mild, moderate and severe TBI with at least a mild level of depressive symptoms	6 months–5 years	5 × 30 min sessions for 10 weeks	1x per week, participants completed a supervised in-clinic exercise session including warm up and cool down. A total of 4x per week, participants performed aerobic exercise on their own or in the community. Participants were asked to maintain 60–80% of their maximal HR.	In clinic and at home	Unspecified	Not reported	Exercise maintenance; mental health; mood; quality of life	Beck Depression Inventory; Medical Outcomes Study 12-Item Short-Form Health Survey; Perceived Quality of Life Scale; 7-day physical activity recall	Depression-like symptoms decreased at 10-weeks, and maintained 6-months later; nearly half of the participants maintained their new activity levels; participants who exercised more had lower depression scores and higher mental health and quality of life

#### 3.5.1 Adults

Eleven of twelve studies employed aerobic interventions at chronic time points post-injury, ranging from 6 months to 23 years (Bhambhani et al., 2005; Brown et al., 2005; Schwandt et al., 2012; Wise et al., 2012; Bellon et al., 2015; Chin et al., 2015, 2019; Weinstein et al., 2017; Ding et al., 2021; Romanov et al., 2021; Tomoto et al., 2022), while one study looked at individuals within 3 months of injury (Morris et al., 2018). Intervention timelines ranged between 4 to 14 weeks, with a minimum of two 15-min weekly sessions (Brown et al., 2005), and a maximum of daily activity (Bellon et al., 2015). Most studies employed supervised interventions, with only three involving a home-based component (Wise et al., 2012; Bellon et al., 2015; Ding et al., 2021). Half of the studies used walking or jogging as the aerobic intervention, typically done on a treadmill (Brown et al., 2005; Bellon et al., 2015; Chin et al., 2015, 2019; Weinstein et al., 2017; Romanov et al., 2021). Depending on the study and participant abilities, some interventions offered physical supports (i.e., walking poles, weight assistance). The other half of the studies used multiple types of exercise or allowed participants to choose their aerobic activity based on abilities and preferences (Bhambhani et al., 2005; Schwandt et al., 2012; Wise et al., 2012; Morris et al., 2018; Ding et al., 2021; Tomoto et al., 2022). Nine of twelve studies tracked participants’ heart rates to ensure aerobic zone adherence, all of which aimed for 60–80% of participants’ maximal heart rates.

Outcomes of interest varied between studies and included measures of mental health, physical health and abilities, cognition, quality of life, carotid arterial compliance, and feasibility of intervention. Eight studies included at least one measure of physical health or ability, all of which reported improvements across their respective interventions, most commonly improvements in VO2 max and heart rates (Bhambhani et al., 2005; Brown et al., 2005; Schwandt et al., 2012; Chin et al., 2015, 2019; Romanov et al., 2021; Tomoto et al., 2022). Six studies included measures related to mental health, with a majority (5/6) reporting improvements in their study-specific measures of stress, depression, quality of life and mood (Schwandt et al., 2012; Wise et al., 2012; Bellon et al., 2015; Weinstein et al., 2017; Chin et al., 2019; Ding et al., 2021). Of note, some studies used self-report for these metrics, while others used standardized inventories (see Table 2). Three studies included cognition-related measures, with Chin et al. (2015) reporting improvements in working memory performance and Romanov et al. (2021) finding improvements in attention. However, Chin et al. (2015) found no changes in cognitive performance across various other cognitive measures from pre- to post-intervention.

These studies should be reviewed with several considerations. First, it is hard to extrapolate if different effects of intervention would be observed if the studies focused on one severity of brain injury, given the heterogenous needs of adults with mild TBI compared to severe TBI. Many studies in this group were pilot studies, and were likely underpowered (Bhambhani et al., 2005; Brown et al., 2005; Schwandt et al., 2012; Chin et al., 2015, 2019; Weinstein et al., 2017; Morris et al., 2018; Ding et al., 2021; Tomoto et al., 2022), requiring follow-up with full studies. Further, several studies did not include control groups at all timepoints (Schwandt et al., 2012; Wise et al., 2012; Bellon et al., 2015; Chin et al., 2015; 2019; Weinstein et al., 2017; Morris et al., 2018; Ding et al., 2021). Intervention adherence also seemed to be a challenge, with adherence rates near 50% in two studies (Bhambhani et al., 2005; Bellon et al., 2015).

### 3.6 Mild brain injury

Ten studies specifically looked at aerobic interventions post-concussion in adults (Leddy et al., 2010; Leddy and Willer, 2013; Polak et al., 2015; Clausen et al., 2016; Adams and Moore, 2017; Dobney et al., 2017; Snyder et al., 2021; Varner et al., 2021; Hutchison et al., 2022; Langevin et al., 2022), and 21 studies focused on adolescents (Gagnon et al., 2016; Imhoff et al., 2016; Chrisman et al., 2017, 2021; Kurowski et al., 2017; Yuan et al., 2017; Chan et al., 2018; Hunt et al., 2018; McGeown et al., 2018; Micay et al., 2018; Bailey et al., 2019; Gladstone et al., 2019; Leddy et al., 2019a,b, 2021; Willer et al., 2019; Dobney et al., 2020; Howell et al., 2021, 2022; Chizuk et al., 2022; Shore et al., 2022). Results for adults are presented in Table 3.

**TABLE 3:** Summary of the effects of aerobic exercise intervention following mild traumatic brain injury in adults.

Refe-rences	Study design	Aim/ objective	Partici-pant details	Time since injury	Intervention timeline	Intervention details	Intervention location	Aerobic modality	Participant adherence	General outcome measures	Specific outcome measures	Results related to aerobic exercise
Adams and Moore, 2017	Case series	To explore changes in outcome measures and return to meaningful life activities in six individuals who had persistent dizziness for at least 9-months post-concussion	*n* = 6; adults aged 18–55; males and females	At least 9 months with dizziness (266–974 days)	Ranging from 14–27 aerobic sessions that occurred for 30 min 3–5x per week; individualized based on participant needs and clinician expertise	60–80% of maximal HR (based on sub-maximal symptom test) aerobic exercise was performed for 30 min 3–5x per week.	Supervised home program	Recumbent cycling	Not reported	Balance; concussion symptoms; dizziness; return to activity; return to work/study	Rivermead Post-concussion Questionnaire Symptoms; Psychosocial impact; Dizziness Handicap Inventory; Activities-specific Balance Confidence Scale; Functional Gait Assessment	Improved Return to work outcomes; all six participants improved their physical activity levels, reduced concussion symptoms, improved dizziness and balance confidence
Clausen et al., 2016	Pre-post-interven-tion study	To evaluate control of cerebral blood flow (CBF) during exercise in females with post-concussion syndrome	*n* = 6; adults aged 23 ± 6; females	6–12 weeks	5–6 × 20 min sessions per week for 12 weeks	HR monitored subsymptom threshold aerobic exercise treatment program at 80% maximum HR from treadmill test.	University concussion clinic	Not specified	Not reported	Concussion symptoms; exercise tolerance	Blood pressure; end-tidal CO2; cerebral bloodflow velocity; minute ventilation; treadmill test; Post-concussion Symptom Scale	Improved exercise tolerance so that symptoms were not exacerbated on the treadmill test; symptoms improved and were not significantly different than the healthy reference group; participants with PCS still had higher HR at onset of exercise than the healthy reference group; PCS participants could only exercise to 90% of their predicted VO2 max even after the intervention; significant increase in min ventilation (Ve) and significant decrease in CO2 partial pressure (PetCO2); significant decrease in CBF velocity
Dobney et al., 2017	Pre-post-interven-tion study	To test the effectiveness of an active rehabilitation program for children and adolescents with persistent post-concussive symptomologies	Aquatic intervention: *n* = 10; adults aged 37.4 ± 15.3; males and females; land control: *n* = 10; adults aged 43.0 ± 14.1; males and females	Pre-intervention appointment: 28 ± 3.3 days; then in-person visit: 40 ± 7.4 days	Daily; number of sessions is dependent on being asymptomatic for 7 days or return to work/play.	Active Rehabilitation protocol. Aerobic activity: stationary bike or treadmill used to reach a target HR zone (50–60%). Resistance is modified until target HR is acquired. Patient exercises for 15 min in target zone.	Location unclear but assumed physiotherapy clinic and home	Stationary bike or treadmill	Not reported	Post-concussion symptoms in four domains: cognitive, emotional, sleep, and physical	Post-concussion Symptom Scale (PCSS) from Sport Concussion Assessment Tool 2 and 3	Active rehabilitation reduced total PCSS score and in all specific domains (cognitive, emotional, sleep, and physical)
Hutchison et al., 2022	RCT	To examine the effect of a structured aerobic exercise program on days to recovery vs. usual care exercise in adolescents and young adults with sports-related concussion	Structured aerobic exercise: *n* = 20; usual care exercise: *n* = 19; adolescent aged 16–22; males and females	3 days	11 sessions over 28 days	Structured exercise program group: 8 × 20 min sessions; 2 days of exercise followed by 1 day rest. Intensity and duration increased each session. Intensity was determined by calculating target HR (progressed 60–75% of age-predicted max HR). Usual care exercise group: followed instructions from sport med physician; subjects advised to increase intensity gradually with minimal head movement then exercises included progression of head movements, visual/cognitive burdens, sport-specific activities, heavy resistance.	Exercise laboratory and at-home; Fitbit monitoring	Stationary bike with limiting movement to head; elliptical or treadmill jogging as alternatives	Not reported but mentioned 6 participants lost to follow up; analyzed *n* = 19 structured exercise group and *n* = 19 usual exercise group	Asymptomatic status; medical clearance; symptom severity	Sport Concussion Assessment Tool 5 (SCAT5); current/premorbid baseline function and objective assessments by physician	Similar symptom severity and total symptom scores at enrollment, but structured aerobic exercise group had lower symptom severity at subsequent assessments vs. usual care group; structured exercise group had faster time to asymptomatic status and earlier medical clearance
Langevin et al., 2022	RCT	To determine if cervicovesti-bular rehabilitation in addition to an aerobic exercise program would reduce mTBI-associated symptoms, as compared to an exercise intervention alone	*n* = 60; adults aged 18–65; males and females	3–12 weeks; mean 39 ± 15 days	Individualized; 8 sessions (30–45 min) over 6 weeks, and asked to continue to follow the program from weeks 6–12	No specific details on the type of aerobic intervention.	Clinic-based; supervised by a kinesiologist	Not specified	Dropout rate was 6.7%; mentioned that “adherence and home exercise was recorded using a self-filled booklet,” but no other mention	Cervical range of motion; clearance to return to function; concussion symptoms; head/neck questionnaires	Post-concussion Symptoms Scale (PCSS); Neck Disability Index (NDI); Headache Disability Inventory (HDI); Dizziness Handicap Inventory (DHI); Numerical Pain Rating Scale (NPRS); Global Rating of Change (GRC); Flexion-Rotation Test (FRT); vestibular/Ocular Motor Screening (VOMS); Head Impulse Test (HIT); cervical segmental motion/sensitivity	Decrease in PCSS, DHI, HDI and BDI; no difference from group receiving cervicovestibular intervention (although this group had better scores on FRT, HIT, range of motion)
Leddy et al., 2010	Case series	To determine if exercise intervention would decrease post-concussion symptoms in a safe manner	*n* = 12; adults aged 27.9 ± 14.3; males and females	6–40 weeks; mean 19 weeks	5–6 days per week for an individualized amount of time (previous treadmill test) or until symptoms increased; treatment was continued until they could complete the treadmill test without increased symptoms	Aerobic exercise at 80% of target HR.	Unclear location; with supervision	Not specified	Not reported, but states compliance was determined by trainer (for athletes) and symptom reports (non-athletes)	Ability to exercise; concussion symptoms, time to recovery	Graded symptom checklist; HR; blood pressure	Decreased symptom score; increased amount of time able to exercise; increased peak HR and systolic blood pressure without symptom increase
Leddy et al., 2013	Pre-post-interven-tion study	To determine if exercise intervention in individuals with post-concussion syndrome would cause a decrease in symptoms, and if their fMRI activity would differ from controls	Concussed: *n* = 8; control: *n* = 4; adolescents and adults aged 17–33; males and females	At most 12 months	20 min per day; 6 days a week; until able to perform at 80% of age-predicted max HR without symptoms	Aerobic exercise at 80% of HR.	At-home or in a gym	Not specified	Not reported	Concussion symptoms; functional magnetic resonance imaging (fMRI) activity; HR	fMRI with math processing test; Post-concussion scale	Increased maximum HR; decreased symptoms; no change in math processing; fMRI activation same as controls with exercise
Polak et al., 2015	Pre-post-interven-tion study	To investigate diffusion tensor imaging characteristics in patients with post-concussion syndrome who received exercise and placebo stretching treatments compared with a group of healthy controls	Control: *n* = 15; adults aged 26.2 ± 1.7; exercise: *n* = 4; adults aged 25.2 ± 5.7; stretching: *n* = 4; adults aged 22.8 ± 6.2; males and females	Exercise: 66 ± 6.6 days; stretching: 170 ± 118.8 days	8 weeks	Controlled and progressive aerobic treadmill test targeted at 80% of this HR, and this program was modified as the HR for symptom aggravation increased. Placebo patients were given instructions for a low-impact breathing and stretching regime and were instructed to keep their HR below 50% of their age predicted maximum. Had to go to Leddy *et al.* to find methods: 20 min per day, 6 days per week.	At-home or in a gym	Treadmill	100%	Diffusion tensor imaging (DTI)	DTI; tract-based spatial stats; potholes	No significance for potholes; reduced number of PCS symptoms and increased maximum HR, but this was not correlated with DTI metrics
Snyder et al., 2021	RCT	To examine adherence, symptom response and key functional outcomes immediately following intervention and at 3 month follow up	*n* = 38; adolescents and adults aged 18–32; males and females	14–25 days	Daily for 7 days; single rest day taken after 3 or 6 days	5 min warm up; 20 min bike at moderate intensity; 5 min break; 20 min bike at moderate intensity; 5 min cool down. Moderate intensity exercise: maintaining 65–75% of estimated HR max, HR max: 208–0.7 × age.	In-person with research staff	Lode Corival stationary bike	Aerobic group attrition: 7.7%; non-aerobic group attrition: 0%	Mood; neurocogni-tion; postural stability; sleep; symptom report	Sport Concussion Assesment Tool 3 (SCAT3); Medical Outcome Scale (MOS); self-reported measure of sleep quality; Beck Depression Inventory (BDI-II); State Trait Anxiety Inventory (STAI); Balance Error Scoring System (BESS); Weschler Adult Intelligence Scale, 3rd ed (WAIS-III); Weschler Memory Scale, 3rd ed; Paced Auditory Serial Addition Test (PASAT); Ruff 2 and 7; DKEFS Trail Making Test; California Verbal Learning Test, 2nd ed (CVLT-II); Letter-number Sequencing; Wisconsin Card Sorting Test; Controlled Oral Word Association	Reduced symptom severity scores; full symptom recovery by 3 month follow up, this did not differ from TBI + non-aerobic group; neurocognitive index scores improved, this did not differ from TBI + non-aerobic group at post-interven-tion or 3 month follow up; positive changes for depression, state anxiety, sleep, and postural stability, not statistically significant between groups
Varner et al., 2021	RCT	To determine if light exercise could prevent post-concussion symptoms at 30 days post injury	Exercise: *n* = 183; control: *n* = 184; adults aged 18–64; males and females	Immediately	48 h of physical rest following the mTBI, followed by daily 30 min aerobic exercise for 1 month	Upon presentation to the emergency room, participants were randomly assigned into a control group, or prescribed light exercise group. Participants were prescribed 30 min of daily aerobic exercise of their choice for 30 days	Home-based	Not specified	Control group: 13 participants withdrew; exercise group: 5 participants withdrew	Post-concussion symptoms	Rivermead Post-concussion Symptoms Questionnaire	No between group differences observed between exercise group and control group on post-concussion symptoms at 30 days, or change in post-concussion symptoms

#### 3.6.1 Adults

Individuals were adults when they sustained their TBIs, with time since injury ranging from 48 h to 2.7 years. Five studies had set intervention lengths (ranging from 1 to 12 weeks), whereas the other studies took an individualized approach to the aerobic intervention duration. The type of aerobic modality performed was only specified in five of the ten studies. Aerobic modalities included recumbent cycling, elliptical and treadmill (Polak et al., 2015; Adams and Moore, 2017; Dobney et al., 2017; Snyder et al., 2021). Participants were assigned a target heart rate in eight of the ten studies, which ranged from 60–80% of maximum. Involvement of a home-based intervention was present in over half of the studies (6/10) (Leddy et al., 2013; Polak et al., 2015; Adams and Moore, 2017; Dobney et al., 2017; Varner et al., 2021; Hutchison et al., 2022), and the majority of studies included supervision throughout the interventions.

All ten studies examined concussion symptoms as an outcome measure. Other outcome measures varied between studies and included return to activity, exercise tolerance, cervical range of motion, head/neck questionnaires, diffusion tensor imaging, cognition, fMRI activity, sleep, and postural stability. Post-concussion symptoms were reduced following aerobic intervention in nine studies (Leddy et al., 2010; Leddy and Willer, 2013; Polak et al., 2015; Clausen et al., 2016; Adams and Moore, 2017; Dobney et al., 2017; Snyder et al., 2021; Hutchison et al., 2022; Langevin et al., 2022). Varner et al. (2021) found no significant change in post-concussion symptoms after 1 month of daily aerobic exercise that began 48 h post-injury compared to the control group. The two studies that examined exercise tolerance found an improvement in participants’ ability to exercise without symptom exacerbation post-intervention (Leddy et al., 2010; Clausen et al., 2016). In relation to brain imaging, Leddy et al. (2013) reported fMRI activation in TBI survivors after exercise to be the same as healthy exercise controls, and Polak et al. (2015) found no correlation between post-intervention symptom reduction and DTI metrics. Of note, both of the aforementioned reports were pilot studies (*N* = 4 concussed individuals in the intervention group), which must be considered when interpreting these data.

Considerations include inadequate comparison groups in some studies (Clausen et al., 2016; Adams and Moore, 2017; Dobney et al., 2017; Langevin et al., 2022). One study provided insufficient details on the aerobic exercise paradigm, and half of the studies did not specify the modality of aerobic exercise performed, affecting their reproducibility.

#### 3.6.2 Adolescents

Most individuals were adolescents (12–18 years) upon sustaining their injury, with time since injury as short as three days up to 2.8 years post-injury (Hunt et al., 2018; Leddy et al., 2019a). Five studies included children (younger than 12 years) (Imhoff et al., 2016; Hunt et al., 2018; Dobney et al., 2020; Leddy et al., 2021; Howell et al., 2022), and two included adults (19 years and older) (McGeown et al., 2018; Howell et al., 2022), but since the average age within the study was in the adolescent range, and no age-separated analyses were done, results are presented in the adolescent section. Studies examining aerobic exercise interventions in adolescents post-concussion can be divided into two categories: early intervention (within 2 weeks post-injury), and chronic intervention for persistent symptoms (4 weeks or longer post-intervention). Due to the large number of studies in this section, the authors sub-grouped and presented the studies based on the above. Full results are presented in Tables 4, 5, respectively.

**TABLE 4:** Summary of the effects of aerobic exercise interventions within two-weeks following mild traumatic brain injury in adolescents.

Refe-rences	Study design	Aim/ objective	Partici-pant details	Intervention timeline	Intervention details	Intervention location	Aerobic modality	Participant adherence	General outcome measures	Specific outcome measures	Results related to aerobic exercise
Chizuk et al., 2022	RCT	To examine whether there is a direct relationship between adherence to a personalized exercise prescription and recovery, or if initial symptom burden affects adherence to the prescription	*n* = 62; adolescent athletes aged 15.77 ± 1.6; males and females	6 × 20 min of aerobic exercise of choice (walking, jogging, stationary cycling) per week for 4 weeks.	Each week, until recovered, participants received a new training target HR based on reassessment of exercise tolerance on the Buffalo Concussion Treadmill Test (BCTT). If a participant did not recover by the 4th week, a more comprehensive form of treatment was initiated. When exercising, participants were prescribed to perform at least 20 min of aerobic exercise of their choice (walking, jogging, stationary cycling) daily for 6 days out of 7, at 90% HR (but this fluctuated). Participants were instructed to stop exercise if their symptoms increased by 2 or more points on a 1- to 10-point visual analog scale when compared with their pre-exercise value.	Home-based	Aerobic exercise of choice (walking, jogging, stationary cycling)	Adolescent adherence rates ranged from 10% to 88%, depending on the week of the intervention; using the definition of completing at least two-thirds of the prescribed volume of aerobic exercise, 31 out of 51 (61%) of the participants were adherent	Cardiorespira-tory response; concussion symptoms	HR; Buffalo Concussion Treadmill Test (BCTT); Post-concussion Symptom Inventory (PCSI)	Those who were adherent were more symptomatic and were more exercise intolerant at their initial visit, yet recovered faster than those who were not adherent; decrease in recovery time as exercise tolerance increased
Dobney et al., 2020	RCT	To examine the feasibility of an active rehabilitation (AR) program for youth with concussion symptoms lasting 2 weeks after injury and compare early AR to usual AR	Early AR: *n* = 10; usual care AR: *n* = 10; children and adolescents aged 9–17; males and females	8 week study period; mean of 3.7 days per week for early AR and 4.2 days per week for usual care AR; mean reported exercise duration of 18.5 ± 11.4 min for early AR and 21.2 ± 21.2 min for usual care AR	Same intervention for early and usual care AR groups. Aerobic exercise at 60% max HR predicted for their age for 15 min.	Home-based with supervision (check-ins after 1-week of intervention)	Aerobic exercise of choice (most common: walking, jogging, stationary cycling)	Participants were more adherent to exercise duration and frequency recommendations; participants were less adherent to the frequency of exercise sessions prescribed (7 days per week)	Concussion symptoms	Post-concussion Symptom Inventory (PCSI)	Symptom severity as measured by PCSI improved over time in both the early and usual care AR groups; 15 of 20 participants had reduced or same symptom severity scores as baseline; early AR group had symptoms that were considered at “normal” (comparable to baseline levels in healthy youth) levels by 4 weeks post-injury, whereas usual care group had normal symptoms at 6-weeks post-injury
Howell et al., 2021	RCT	To examine the effects of a prescribed aerobic exercise program on symptom severity and exercise volume	*n* = 41; adolescents and adults aged 14–21; males and females	8 week aerobic exercise; 5x per week; 20 min at target HR	Provided aerobic exercise prescription (intervention group: intensity, duration, frequency) within 14 days of injury. At baseline, participant aerobic fitness was determined and initial exercise prescription was based on results; individualized target HR, frequency (5x per week); 20 min at target HR. At 4-weeks post-enrollement underwent aerobic exercise test and prescription intensity was adjusted as needed but same volume (20 min per day, 5x per week) and symptoms were assessed. Standard-of-care group adhered to physician recommended physical activity. Modes of exercise were left to participant preference. Weekly log of aerobic activity; reported symptom severity at 1-and 2-months post-enrollement. Subjects were given HR monitor to ensure exercised at prescribed intensity and to monitor daily activity.	Unreported; implied gym/home or other since mode of exercise was left up to participant	Not specified	41 enrolled; 37 included in analysis; 4 excluded because they did not complete >50% of exercise diaries	Exercise volume; symptom severity	Post-concussion Symptom Inventory (PCSI); exercise volume (average min/week); must have completed >50% of exercise diaries	No significant differences in symptom severity between prescription vs. standard-of-care groups; exercise volume was also similar; greater exercise volume was associated with lower symptom burden after 1 month (i.e., those with <100 min/week exercise had higher symptom severity regardless of group); exercise volume of >160 min/week was associated with symptom resolution after 1 month
Howell et al., 2022	RCT	To determine the proportion of adolescents at moderate or high risk of PPCS that develop PPCS when prescribed early aerobic exercise vs. standard-of-care and examine exercise volume	*n* = 16; children and adolescents aged 10–18; males and females	Five × 20 min sessions per week for four weeks	Early aerobic exercise group: individualized target intensity (80% of HR at end of exercise test) and uniform volume (5x/week for 20 min/day). Standard-of-care group: adhere to physician’s recommendations on physical activity; symptom limited activity without specific exercise recommendation.	Unreported; implied at-home	Not-specified	100%	PPCS; exercise volume and intensity	Post-concussion Symptom Inventory (PCSI) at 1-month post-concussion; Average min/week recorded aerobic exercise, Average HR and max HR recorded during each session	Smaller proportion of early aerobic exercise group developed PPCS compared to standard-of-care group. Exercise volume and intensity were not significantly different.
Leddy et al., 2019a	RCT	To determine if daily exercise would decrease the time to recovery and symptoms following a sports-related concussion in adolescent athletes	*n* = 103; adolescent athletes aged 13–18; males and females	Daily for 20 min or until symptoms increased; intervention was until they recovered from their injury as determined by the physician, or after 30 days	Aerobic exercise at 80% of target HR; participants were not allowed to stretch before or after exercise.	At-home or in a gym; with supervision	Stationary bike; treadmill; walk/jog	89% of daily reports included completion of exercise intervention that day; 7 participants were removed because they missed 3 days in a row or completed less than 75% of daily symptom reports	Concussion symptoms; days to recovery	Post-concussion Symptom Scale	Exercise decreased the time to recovery; no significant change in symptoms
Leddy et al., 2019b	Pre-post-interven-tion study	To determine if exercise intervention would decrease the time to recovery and concussion-related symptoms in male adolescent athletes	*n* = 54; adolescent athletes aged 13–18; males	Daily for 20 min or until symptoms increased for 14 days	Aerobic exercise at 80% of target HR; 5 min warm-up and 5–10 min of cool-down.	At home or in a gym; with supervision	Stationary bike; treadmill	93.2% of daily symptom scores were completed (6.8% absent)	Concussion symptoms; days to recovery	Post-concussion Symptom Scale	Exercise decreased recovery time from first visit and symptom scores after 14 days
Leddy et al., 2021	RCT	To determine if exercise intervention would reduce recovery time and symptoms from a sport-related concussion, including safety and adherence	Adolescents: *n* = 38; aged 9–18; males and females; parents: *n* = 38	Daily for 20 min for up to 4 weeks or until recovery	Aerobic exercise at up to 90% of target HR.	Home-based	Walking; jogging; stationary bike	Exercise: 65%; control: 50%	Concussion symptoms; exercise ability; recovery time	Post-concussion Symptom Inventory; HR	Exercise decreased recovery time and resulted in fewer symptoms; increased HR during exercise and amount of time exercising per day
Micay et al., 2018	RCT	To examine the feasibility of implementing a standardized aerobic exercise intervention in the post-acute stage of sport-related concussion recovery in a sample of adolescent students with sport-related concussion compared with usual care	Intervention: *n* = 8; control: *n* = 7; adolescents aged 14–18; males	Approximately 4–6 weeks until time to clearance of return to sport	8 sessions proceeding in a stepwise fashion with respect to duration and intensity using the Velotron Racermate Pro stationary cycle ergometer; 50–70% age-predicted max HR (intensity increased over sessions). A total of 20 min sessions.	University concussion clinic	Recumbent cycling	100%; 1 participant excluded because she was the only female	Return to play; symptom severity	Efficacy of the intervention; symptom status (Post-concussion Symptom Scale); time to medical clearance	No change in time to clearance; significant correlation between acute symptom severity and overall time to medical clearance
Willer et al., 2019	Pre-post-interven-tion study	To compare outcomes of adolescents with concussion who were prescribed rest, aerobic exercise or stretching	Exercise: *n* = 52; stretching: *n* = 51; rest *n* = 48; adolescents aged 13–18 years; males and females	Daily 20 sessions for 4 weeks or until recovery, whichever came first	Participants warmed up for 5 min, performed aerobic exercise (walking, jogging or biking), for 20 min, and cooled down for 5 min. Participants were told to stop exercising if their symptoms got worse. All exercise was sub-threshold, calculated as 80% of the HR achieved at symptom exacerbation or voluntary exhaustion.	At-home or in-clinic	Aerobic exercise of choice	7 participants lost to follow up; 10 participants removed because they did not report daily symptoms; 1 participant had influenza and was removed	Concussion symptoms; days to recovery	Sport Concussion Assessment Tool 3 (SCAT3); Post-concussion Symptom Scale	Exercise group recovered faster than the rest group by an average of 3 days; no between group differences in delayed recovery; female participants in the rest group showed an acute increase in symptoms compared to the other groups

**TABLE 5:** Summary of the effects of aerobic exercise interventions implemented four weeks or longer following mild traumatic brain injury in adolescents.

Refe-rences	Study design	Aim/ objective	Partici-pant details	Intervention timeline	Intervention details	Intervention location	Aerobic modality	Participant adherence	General outcome measures	Specific outcome measures	Results related to aerobic exercise
Bailey et al., 2019	RCT	To describe a model multidisciplinary concussion management and explore management methods in the acute and post-acute settings	Intervention: *n* = 7; control: *n* = 8; adolescents aged 14–18; males and females	Daily 20 min sessions per week for 6 weeks	80% of individual maximum HR threshold.	3 supervised clinic sessions per week, and home-based exercise between clinic days	Treadmill running	Not reported	Concussion symptoms; neuropsycho-logical; postural stability	Post-concussive Scale Revised (PCS-R); Beck Depression Inventory	Participants in the exercise group had less symptoms at follow-up
Chan et al., 2018	RCT	To examine the safety and tolerability of an active rehabilitation program for adolescents who are slow to recover from a sport-related concussion	Intervention: *n* = 10; control: *n* = 9; adolescents aged 15.9 ± 1.66; males and females	Mean 3.4 sessions per week for 6 weeks (not including at home exercise program)	Consisted of submaximal aerobic training, light coordination and sport-specific exercises, visualization and imagery techniques, and a home exercise program.	Outpatient concussion clinic and home-based	Not specified; mentioned light aerobic exercise	100%	Cognitive performance; fatigue; measures of health-related quality of life; mood; self-reported post-concussion symptoms	Post-concussion Symptom Scale (PCSS); Patient-Reported Outcomes Measurement Information System; Beck Depression Inventory for Youth–Second Edition; Pediatric Quality of Life Multidimensional Fatigue Scale; Teen Report Standard Version; Balance Error Scoring System; Immediate Post-concussion Assessment and Cognitive Test	Statistically significant treatment effect on post-concussion symptoms; no adverse effects when compared with control group
Chrisman et al., 2017	Pre-post-intervention study	To examine the safety of a Sub-Symptom Threshold Exercise Program (SSTEP) in youth and determine if there is a correlation with concussion symptom improvement	*n* = 87; children and adolescents aged 14.9 ± 2.3; males and females	Daily sub-threshold physical activity for the same time they could perform physical activity during the initial treadmill testing (or for a maximum of 20 min). A total of 1–2 week follow up and continued the exercise intervention until they could complete the Balke treadmill test without worsening symptoms	Initial evaluation with the Balke treadmill test to obtain a heart-rate threshold above which they report increased symptoms. Prescribed an at home sub-threshold exercise program (at a HR 80% of that that causes symptoms).	Home-based	Treadmill for the initial determination of HR threshold; no specific details on the prescribed at-home component	142 patients referred to SSTEP program; 83 underwent treatment meeting inclusion criteria	Concussion symptoms	Sport Concussion Assessment Tool 2 (SCAT2)	SCAT2 scores decreased exponentially over time after beginning the intervention; no participant had symptoms worsen after beginning SSTEP; monitored exercise from the SSTEP program was safe for youth and there was a correlation with improvement in concussion symptoms
Chrisman et al., 2021	Pre-post-intervention study	To assess feasibility and acceptability of a telehealth delivered exercise intervention for concussion, the Mobile Subthreshold Exercise Program (MSTEP), and collect pilot data regarding efficacy	*n* = 19; adolescents aged 14.2 ± 2.2; males and females	7x exercise (at home choice) per week for 6 weeks	Initial goal was set at 10 min at a HR of 120. Individuals could choose the type of exercise they completed. If symptoms worsened during exercise, youth were instructed to take a break and decrease the HR goal utilized until they were able to tolerate 10 min of exercise. Goals were advanced weekly as tolerated to a maximum of 60 min of physical activity per day at a HR of 140. Participants would wear Fitbits and fill out surveys.	Home-based	Aerobic exercise of choice at home, as long as HR was increased to goal (120 or 140)	1 individual withdrew from the study at 3 weeks due to increasing headaches; participants wore the Fitbit on 80% of days and completed 94% of surveys and 96% of Zoom calls	Concussive symptoms; health-related quality of sleep; symptoms of anxiety and depression	Health and Behavior Inventory; Pediatric Quality of Life Inventory; Fear of pain questionnaire (adapted for concussive symptom); Patient Health Questionnaire-9; Generalized Anxiety Disorder Scale-7; Adolescent Sleep Wake Scale-10 item	Concussive symptoms improved significantly from baseline to weeks 3 and 6; health-related quality of life improved
Gagnon et al., 2016	Pre-post-intervention study	To investigate an active rehabilitation (AR) program in adolescents who experienced persistent post-concussive symptomo-logies following sports related concussion	*n* = 10; adolescents aged 14–18; males and females	Daily; number of sessions dependent on being asymptomatic for 7 days; treatment lasted 6.8 ± 4.7 weeks post-injury	15 min of aerobic training at 60% of maximal capacity.	Clinic-based and home program	Stationary bike or treadmill	Not reported	Post-concussion symptoms	Post-concussion Scale; mood (Beck Depression Inventory); energy level (Pediatric Quality of Life Multidimensional Fatigue Scale); balance and coordination (body coordination component of Bruininks-Oseretsky test of motor proficiency); cognitive function (ImPACT)	(Reduced post-concussion symptoms on the post-concussion scale); increased processing speed (ImPACT); no changes on other measures
Gladstone et al., 2019	RCT	To determine the if subsymptom aerobic exercise improves quality of life and cognition following mild TBI	*n* = 30; adolescents aged 12–17; males and females	6–8 weeks; 5–6 sessions a week	At baseline participants Borg rate of perceived exertion of 11 (resistance level of 2) for 5 min. Perceived exertion was increased every 5 min for 30 min; during intervention participants exercised 5–6 times per week at 80% of the level that exacerbated symptoms. Exercise levels were modified with 6 visits.	At-home and in-clinic	Upright exercise bike	Participants completed 4.42 ± 1.95 sessions per week	Cognition; quality of life	NIH toolbox Cognition Battery (Fluid and Crystalized Cognition Composite Score); PedsQL Generic Core	Increased Fluid and Crystalized Cognition Composit scores; Increased PedsQL scores
Hunt et al., 2018	Pre-post-intervention study	To identify key components in an active rehabilitation program and receive perspective from youths with mTBI and their parents	Adolescents: *n* = 38; aged 9–18; males and females; parents: *n* = 38	6 week active rehabilitation program; 1x daily	Prescribed home program tailored to individual. A total of 4 components: (1) low intensity aerobic exercise for 15 min (treadmill or stationary bike), (2) sport specific coordination drills (max 10 min), (3) relaxation (deep breething, visualization) for 5 min, (4) concussion education/support (initial 1 h session and ongoing support/reinforcement through phone calls). Education topics included: concussion awareness, sleep, nutrition, energy management, return to school and return to play.	Home-based	Treadmill or stationary bike	38 youth and 36 parents completed post-intervention survey	Parent experience; youth experience	Survey measured whether active rehabilitation program was helpful; which strategy aided in concussion recovery; which strategy they’d recommend to a friend; program adherence	100% youth reported active rehab was helpful; top 3 helpful strategies reported were: energy management strategies (47.4%); aerobic exercise (31.6%), and sports drills (21.1%); key ingredients: learning energy management, physical activity, seeking help; 100% parents would recommend program; key ingredients: encouraging recovery through structured activities, youth accountability, patience/accepting uncertain timelines
Imhoff et al., 2016	Pre-post-intervention study	To identify if active rehabilitation intervention influences recovery of slow-to-recover mTBI patients	*n* = 18; youth aged 10–17; males and females	3 × 15–45 min sessions per week for an unlimited amount of weeks until symptom-free status was reached (48 ± 88 days)	The intervention had 3 components: (1) progressive sub-maximal low- to high-intensity (based on rate of perceived exertion) aerobic cycling for up to 20 min; (2) low intensity sport specific coordination exercises up to 10 min; (3) therapeutic balance exercises.	Supervised home programme	Recumbent cycling	85% ± 20%	Balance; coordination; concussion symptoms; neuropsycholo-gical; return to play	Neuropsycholo-gical: RAVLT; Verbal Fluency; Digit Span; SDMT; CPTII	Decreased concussion symptoms; improved verbal memory; semantic fluency and working memory; improved balance
										Coordination and Balance: SCAT3; Biosway; BOT2	
Kurowski et al., 2017	RCT	To examine the effects of aerobic training for the management of prolonged symptoms in adolescents with mTBI	Aerobic training: *n* = 15; stretching: *n* = 15; adolescents aged 12–17; males and females	5–6 days per week for at least 6 weeks (+ up to 2 weeks); participants that did not return to preinjury symptom level continued in their program for up to two additional weeks	Cycling group: tailored sub-symptom exacerbation aerobic exercise program based on aerobic cycling test performance at baseline; 80% of the duration that exacerbated symptoms at baseline assessment. Reassessment performed weekly and aerobic program adjusted accordingly. Stretching group: full-body stretching program that targeted upper and lower extremities and mid-section; rotated on biweekly basis.	Home-based	Cycling	Adherence lower in aerobic training group (4.42 times per week) vs. stretching (5.85 times per week)	Symptom severity	Post-concussion Symptom Inventory (PCSI); self-ratings considered primary outcome (pre-injury; pre-intervention), at interval visits, and after run-out period	Greater rate of improvement based on self-reported PCSI in aerobic training group vs. stretching
McGeown et al., 2018	Pre-post-intervention study	To evaluate the effect of exercise-based rehabilitation on symptom scores, brain-derived neurotrophic factor (BDNF), cognitive functions and static balance in a sample of participants with post-concussion syndrome	*n* = 9; adolescents and adults aged 14–21; males and females	4 weeks (3 sessions per week)	Warm-up followed by, stationary cycling, static balance training, and then cool down exercises requiring approximately 40–60 min for each session. Balance exercises included the same three positions performed during the balance error scoring system (BESS) protocol; DS with feet side by side and touching; SL while standing on one leg; and TS with the heel of one foot directly in front of the toes of the other foot. Participants completed three sets of each exercise, with a one min rest between each exercise.	University concussion clinic?	Recumbent cycling	100%, but some participants took up to 78 days to complete the study because a session would be stopped if it exacerbated their concussion symptoms	Balance; cognitive function; concussion symptoms; salivary-BDNF levels	Salivary BDNF	No statistically significant differences for resting HR, resting systolic BP, resting diastolic BP, or for salivary-BDNF concentrations following the aerobic and balance exercise program; no other significant results mentioned related to aerobic exercise
Shore et al., 2022	Pre-post-intervention study	To explore the feasibility of a newly developed, remote accessible active rehabilitation program (Tele-Active Rehabilitation) that was designed for adolescents with concussion	*n* = 3; adolescents aged 14–17; males and females	3 days per week for 6 weeks	10–30 min of subsymptom threshold activity, progressed throughout the program. Started 10 min at level 4 Pictorial Children’s Effort Rating Table (PCERT), if tolerated progressed to no more than 30 min at level 6 PCERT.	Gym or at-home	Walking; running; cycling; swimming	77–100%	Illness perception; occupational performance; post-concussion symptoms; program satisfaction	Post-concussion Symptom Inventory (PCSI)–adolescent version; Brief Illness Perception Questionnaire (BIPQ); Canadian Occupational Performance Measure (COPM); Client Satisfaction Questionnaire (CSQ)	Decreased post-concussion symptoms (PCSI); most pronounced symptom reduction from week 0 to week 3; lower BIPQ scores indicating more favorable perception of their condition; 2 of 3 participants reached clinically significant change in COPM score (other approached clinical significance) indicating better occupational performance
Yuan et al., 2017	RCT combined with case-control comparison	To determine structural connectivity changes after aerobic exercise training in adolescents with persistent symptoms after mTBI	Exercise: *n* = 8; stretching: *n* = 9, case-control participants selected from pediatric functional neuroimaging research network; mean age 15.45 ± 1.72; males and females	5–6 days per week for at least 6 weeks (+ up to 2 weeks); participants that did not return to preinjury symptom level continued in their program for up to two additional weeks.	Cycling group: tailored sub-symptom exacerbation aerobic exercise program based on aerobic cycling test performance at baseline; 80% of the duration that exacerbated symptoms at baseline assessment. Reassessment performed weekly and aerobic program adjusted accordingly. Stretching group: full-body stretching program that targeted upper and lower extremities and mid-section; rotated on biweekly basis.	Home-based	Cycling	Not reported	Concussion symptoms; structural connectivity	Global efficiency; mean local efficiency; modularity; normalized clustering coefficient; normalized characteristic path length; small-worldness; Post-concussion Symptom Inventory score	Increased global efficiency and decreased normalized characteristic pathlength in exercise group; improved concussion symptoms were correlated with increase global efficiency in the aerobic group

##### 3.6.2.1 Adolescents—Acute timepoint

Nine studies assessed adolescents with mTBI within 2-weeks of injury (64; 54,55,57; 89; 25; 37, 39; 18). Intervention timelines ranged from 4 to 8 weeks; however, several studies discontinued the intervention once return-to-play status was assumed. Most commonly, studies had participants engaging in daily aerobic exercise for 20 min (54,55,57; 89), or 20-min sessions 5 days per week (37, 39). The remaining studies examined 6x per week, 3x per week, 2x per week or personalized frequency for 15–20 min per session (64; 25; 18). All studies tracked heart rate and had participants exercising at 60–90% of their max heart rates during the exercise sessions. Eight studies allowed participants to choose their aerobic modality, if heart rate targets were met; most commonly participants engaged in jogging, walking or recumbent cycling. 64 had participants exclusively use recumbent cycling as an aerobic activity.

These particular studies showcased a distinct set of outcome measures. All studies were primarily centered on recovery from concussion, frequently evaluating parameters such as the duration of recovery, concussion symptoms, time taken to return to play, and participants’ ability to tolerate exercise. Of the eight studies that evaluated recovery time, seven found that implementing early exercise interventions reduced time to recover compared to standard care (Leddy et al., 2019a,b, 2021; Willer et al., 2019; Dobney et al., 2020; Howell et al., 2021; Chizuk et al., 2022). Additionally, Howell et al. (2022) reported that the early exercise group was less likely to develop persistent post-concussion symptoms compared to the usual treatment group.

Adherence to the exercise interventions ranged from 61 to 100% across studies, and interestingly, Chizuk et al. (2022) reported that adherent participants recovered faster than non-adherent participants, supporting the benefits of early exercise post-mTBI. While this set of studies represents the most cohesive group, most studies focus on sport-related concussions, therefore, these results may not be generalizable to wider populations.

##### 3.6.2.2 Adolescents—Chronic timepoints

Twelve studies examined aerobic exercise for adolescents at least 1 month post injury who were experiencing at-least one persistent post-concussion symptom (Gagnon et al., 2016; Imhoff et al., 2016; Chrisman et al., 2017, 2021; Kurowski et al., 2017; Yuan et al., 2017; Chan et al., 2018; Hunt et al., 2018; McGeown et al., 2018; Bailey et al., 2019; Gladstone et al., 2019; Shore et al., 2022). A majority of these studies offered a mix of in-clinic and at-home interventions for 6 weeks (Chan et al., 2018; Hunt et al., 2018; Bailey et al., 2019; Chrisman et al., 2021; Shore et al., 2022), with up to an additional two weeks, making an 8-week intervention (Kurowski et al., 2017; Yuan et al., 2017; Gladstone et al., 2019). One study consisted of a four-week intervention (McGeown et al., 2018). In contrast, the remaining three studies offered the intervention until symptom resolution occurred (Gagnon et al., 2016; Imhoff et al., 2016; Chrisman et al., 2017). Intervention frequency ranged from 3 times per week (15–45 min) to 20 min daily. Ten studies included effort-based measures (e.g., Borg scale) or physiological measures (i.e., heart rate) to gauge aerobic status during exercise (Gagnon et al., 2016; Imhoff et al., 2016; Chrisman et al., 2017, 2021; Kurowski et al., 2017; Yuan et al., 2017; Chan et al., 2018; Bailey et al., 2019; Gladstone et al., 2019; Shore et al., 2022). Recumbent cycling was the exclusive aerobic modality in five studies (Imhoff et al., 2016; Kurowski et al., 2017; Yuan et al., 2017; McGeown et al., 2018; Gladstone et al., 2019), while five studies offered participants their choice of activity (Gagnon et al., 2016; Chan et al., 2018; Hunt et al., 2018; Chrisman et al., 2021; Shore et al., 2022). Two studies had participants engage in treadmill-based walking or running (Chrisman et al., 2017; Bailey et al., 2019).

While nine of twelve studies in this set examined concussion-related symptoms, many included additional measures of mental health, mood, cognition, intervention experience, balance, saliva-based protein analysis, illness perception and structural connectivity. A total of 100% of studies that examined concussion-related symptoms found improvements post-intervention; however, only five of these studies included appropriate comparison groups (Gagnon et al., 2016; Imhoff et al., 2016; Chrisman et al., 2017, 2021; Kurowski et al., 2017; Yuan et al., 2017; Chan et al., 2018; Bailey et al., 2019; Shore et al., 2022). In terms of cognition, Gladstone et al. (2019) found that exercise increased crystallized and fluid cognition composite scores, Gagnon et al. (2016) reported improved processing speed and no differences in balance, while Imhoff et al. (2016) reported improvements in multiple memory tasks and balance. McGeown et al. (2018) examined salivary brain-derived neurotrophic factor (BDNF), but did not find any improvements post-exercise. However, the authors suggest this can be attributed by the large variance in baseline levels and the variable intervention length. One study examined functional connectivity and found that participants in the exercise group had increased global efficiency and decreased normalized path length (Yuan et al., 2017). Only one study assessed attitudes and feasibility of the exercise intervention; 100% of the participants reported that the active rehabilitation was effective (Hunt et al., 2018).

Many considerations are prudent when assessing this group of studies. First, the sample sizes in ten of the twelve studies are below 30 individuals, which may indicate that a majority of these studies are underpowered. Additionally, only five studies included control or comparison groups, which helps determine effects related to the intervention.

### 3.7 Unspecified

Five studies did not specify the severity of traumatic brain injury in participants undergoing aerobic interventions, but referred to trauma-induced brain injury (Hoffman et al., 2010; Esquenazi et al., 2013; Kolakowsky-Hayner et al., 2017; Pennington et al., 2022; Tefertiller et al., 2022). Full results are presented in Table 6.

**TABLE 6:** Summary of the effects of aerobic exercise interventions following unspecified-severity traumatic brain injury.

Refe-rences	Study design	Partici-pant details	TBI demo-graphics	Time since injury	Intervention timeline	Intervention details	Intervention location	Aerobic modality	Participant adherence	General outcome measures	Specific outcome measures	Results related to aerobic exercise
Esquenazi et al., 2013	RCT	Robotic-assisted: *n* = 8; adults aged 37.1 ± 10.6; manually assisted: *n* = 8; adults aged 41.9 ± 16.8; males and females	Severity not specified but participants had issues with ambulation as the result of a TBI	At least 1 year	6–8 weeks; 3x per week for 18 sessions; 45 min each session	Gait Mat with self-selected velocity and maximal velocity set at 10–20% of body weight support. Every 3rd session the self-selected velocity and maximal velocity were assessed and if either measure improved the training speed was increased (maximum of 10%). Manually assisted locomotor therapy group used a Lite Gate body weight support system and therapist support. The robotic-assisted locomotor therapy group with a Lokomat.	Clinic-based	Treadmill	1 participant from manually assisted treadmill group withdrew	Gait parameters	Self-selected velocity and maximal velocity; secondary gait measurements with the 6-Min Walk Test and the mobility domain of Stroke Impact Scale	Self-selected velocity and maximal velocity increased in both robotic-assisted and manually assisted treadmill training groups; no differences between the two groups; for 6-Min Walk Test no change in robotic-assisted therapy but an increase in manually assisted therapy; for stroke impact scale, increase in both groups
Hoffman et al., 2010	RCT	Control *n* = 40; treatment *n* = 40; adults aged 37–40; males and females	Self-reported TBI severe enough to require medical intervention/ hospital admission; score of ł5 on Patient questionnaire-9; must report at least mild depression severity at baseline	6 months–5 years	10 weeks; 1x per week	Each session included 15 min warm-up (stretching and walking); 30 min aerobic exercise (incline treadmill, stair-stepper, rowing machine, stationary bike, indoor track). Exercise intensity was adjusted to reach HR goal that was 60% estimated maximum HR (220-age), which would have participant exercise approx 50% of their aerobic capacity and reach perceived exertion of 12 on a 20-point scale; 15 min of cool down (stretching, slow walking). Participants were also asked to perform additional 30 min of aerobic exercise 4x per week at home.	Community gym and additional aerobic exercises at-home	Treadmill; stair-stepper; rowing machine; stationary bike; indoor track	84 enrolled; 4 did not attend sessions after enrollment; 76 participants completed 10-week assessment; unable to contact 4 subjects for 10 week assessment; mean number of sessions attended was 5.88 where more than half exercise group attended 7 or more sessions	Severity of depression	Beck Depression Inventory (BDI); physical symptoms of depression/ health; pain; fatigue; Brief Pain Inventory (BPI); Pittsburgh Sleep Inventory; Head Injury Symptom checklist; SF-12 Health Survey	No significant differences on the BDI (severity of depression) in treatment vs. control; exercise group had less pain interference and greater improvement on BPI compared to control; no improvement on increasing min of exercise, head injury symptoms, perceived quality of life, sleep, general health, HR, or ability to walk; high activity sub-group (greater than 90 min exercise per week) had lower depression scores than low activity group; high activity group also reported more community activity, better quality of life, better general mental health.
Kolakowsky-Hayner et al., 2017	RCT	Walking first: *n* = 62; nutrition first: *n* = 63; adults aged 42.7 ± 15.5; males and females	TBI at least 6 months prior that required medical attention; must be able to ambulate; use of orthotics, cane/walker permitted	6 months	36 weeks total; 12 weeks exercise period; 12 weeks nutrition period; 12 weeks washout	Use pedometer to track steps; total number of steps, aerobic steps, calories burned, and miles walked. Assigned coach set time, weight and stride length. Goal of 5% increase over individual baseline in daily steps for first week. Subsequent weeks, step goal was increased until reached 40% incease in week 8 and maintained in last 4 weeks.	Home-based	Walking	Poor adherence; almost a third discontinued intervention	Fatigue	Global Fatigue Index (GFI); the Barrow Neurological Institute (BNI) Fatigue Scale Overall Severity Index Score; and the Multidimen-sional Fatigue Inventory (MFI); domains of fatigue: severity, distress, impact on activity, and timing of fatigue	Exercise-first and nutrition-first groups increased number of steps over time; less fatigue (GFI, BNI total and MFI general score) after walking intervention, whereas nutrition intervention had little impact on fatigue; less fatigue after 24 weeks (active part of intervention) and wash out period (36 weeks)
Tefertiller et al., 2022	RCT	*n* = 31; adults aged 18–65; males and females	Chronic TBI with self-reported and objective balance deficits, completed inpatient rehabilitation at single TBI specialized rehabilitation hospital	At least 1 year	1 h sessions; 3x per week for 4 weeks	5 min of balance training; >35 min of mobility training; Virtual Reality + Treadmill Training (VRTT) group: Treadmill training with feedback on gait in the form of VR games; Treadmill Training (TT) group: Treadmill training only.	Clinic-based; supervised by physiotherapist	Motek C-Mill Treadmill	100%	Balance; mobility	Community Balance and Mobility Scale (CB and M); 10 meter walk test (10MWT); 6 min walk test (6MWT); Timed Up and Go (TUG); HR; perceived exertion; Physical Activity Enjoyment Scale (PACES)	Improved CBand M; nominal increases 10MWT; increased 6MWT; nominal improvements in TUG; VRTT and TT groups had greater PACES score indicating more enjoyment of the program compared to standard care group; no significant differences relating to aerobic exercise and current standard of care; all groups had improved balance and mobility after 12 week intervention and at 4 week follow up
Pennington et al., 2022	Pre-post-interven-tion study	Exercise only: *n* = 13; gameplay control: *n* = 12; VR-EFT: *n* = 18; males and females	Not reported, but all had alcohol use disorder	At least 6 months	8 weeks	Used a recumbent bicycle, a set of gameplay controllers mounted on the recumbent bicycle handlebars, and an Oculus Rift VR headset. A total of 3 sessions of control (exercise or game) per week for 3 weeks, 1 week rest, 3 weeks combined game while on bike, 3x per week.	San Francisco Veterans Affairs Health Care System	Recumbent cycling	7/30 lost to follow up for various reasons; 7/30 did not do 8 week follow up (mostly due to COVID); 23/30 included in analysis; overall 53% completed the study	Neuropsycho-logical testing; PCS; alcohol use and craving	Working memory; visual scanning; cognitive flexibility; cognitive inhibition; cognitive inhibition-switching; processing speed; auditory-verbal immediate recall; auditory-verbal delayed recall; visuospatial immediate recall; visuospatial delayed recall; exploratory analyses: within group change in alcohol use, craving and post-concussive symptoms; drinks per week; Obsessive Compulsive Drinking Scale (OCDS) total; OCDS Obsessive Subscale; OCDS Compulsive Subscale; Neurobehavioral Symptom Inventory	Combined exercise and game had improvement in cognitive inhibition switching and visual scanning; significant improvement in cognitive inhibition associated with the exercise-only condition; no changes in alcohol consumption following the combined intervention; no changes in post-concussion symptoms in any intervention group.

All participants in these studies were adults, with time since injury ranging from 6 months to 5 years. Aerobic exercise interventions spanned from 4 to 12 weeks. Two of these interventions included home-based components and the other three were clinic-based. Aerobic modalities varied amongst studies, including the use of treadmills, stationary cycling, rowing machines, stair-steppers, indoor tracks, and outdoor walking. Four studies used a single modality of aerobic exercise, three of which had participants walking (two on the treadmill, one with home-based walking) and one study used stationary cycling (Esquenazi et al., 2013; Kolakowsky-Hayner et al., 2017; Pennington et al., 2022; Tefertiller et al., 2022). The study using stationary cycling also involved a combined intervention with virtual reality (Pennington et al., 2022).

Outcome measures assessed in these studies were gait, severity of depression, fatigue, balance, mobility, neuropsychological assessments, alcohol use, and post-concussion symptoms. Esquenazi et al. (2013) found that participants improved their mobility, along with self-selected and maximum velocity on the treadmill after the aerobic exercise intervention. Hoffman et al. (2010) found an improvement in pain after the exercise intervention, and lower post-intervention depression scores for the high-activity exercise group compared to the low-activity group. Participant fatigue was reduced after intervention in Kolakowsky-Hayner et al. (2017) and Tefertiller et al. (2022) found improved balance and mobility post-intervention which persisted at the 4-week follow-up. Improved cognitive inhibition was reported by Pennington et al. (2022); however, they found no change in alcohol use or post-concussion symptoms post-intervention.

It should be noted that there was low adherence to the home-based intervention in Kolakowsky-Hayner et al. (2017), with almost a third of participants discontinuing the intervention. Pennington et al. (2022) only had 53% of participants fully complete their in-person study, which included an 8-week follow-up. Additionally, target heart rate zones were only assigned to participants in one of the five studies (Hoffman et al., 2010).

## 4 Discussion

Fifty-four studies were included in this review, and first divided based on injury severity (i.e., severe, mixed, mild, unspecified), then subdivided based on age (i.e., adult, adolescent), and time since injury (i.e., acute, chronic) when applicable. The purpose of this review was to describe the breadth of aerobic interventions used to improve health-related outcomes post-TBI across the lifespan. However, it is worth noting that no studies exclusively examined aerobic exercise following moderate TBI, nor in children younger than 12, and therefore these groups are absent from the present review.

For adults, aerobic exercise post-TBI is a promising intervention, especially as no studies reported adverse effects for participants. However, it is worth considering how the impact of aerobic exercise may be influenced by factors such as the severity of the injury and time since injury. In studies focusing on severe brain injury, aerobic exercise seems to be effective at improving cardiovascular health and balance in TBI survivors, and shows promise for improving cognitive functions, like memory, which is supported by increased hippocampal volume post-intervention. In mixed and unspecified injury severity groups, again, aerobic exercise shows benefits related to cardiovascular and physical and fitness, cognition, and mental health. The studies focused on mild TBIs, including concussions, showed more consistent benefits for aerobic exercise interventions, particularly in symptom reduction and exercise tolerance. However the lesser-studied effects of these interventions on cognitive and neuroimaging measures are mixed. Overall, the body of evidence generally supports aerobic exercise as a safe, easily implementable intervention for adults post-TBI.

In adolescents, 21 studies examined aerobic exercise post-mild traumatic brain injury, and one study presented a case study post-severe TBI. From this cohort of studies, aerobic exercise is a safe and beneficial intervention for post-concussion adolescents, and can be effectively implemented as early as one-week post-injury. Overall, early implementation seems to promote recovery compared to standard care, and chronic implementation is effective at reducing concussion-related symptoms and supporting cognition and fitness.

**FIGURE 3:**
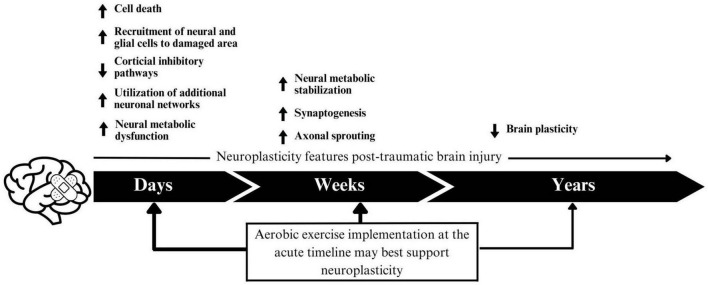
A proposed schematic of negative plasticity following traumatic brain injury. Arrow thickness indicates the proposed impact of intervention within that timeframe.

While these findings are promising, there are several notable limitations in the present study and existing research. In the present study, while we aimed to conduct a comprehensive review, we could not include papers that did not have an official English translation. In the literature, although many studies showed positive results of aerobic exercise, an individual’s time since injury should be considered. Time since injury can play a role in physical recovery. For example, with severe brain injury, multiple physical injuries are common activity-limiting comorbidities (Chan et al., 2017). Several studies included assisted-physical activity, by use of walking supports, physical therapists present, and including non-balance dependent exercise (e.g., hand cranking), demonstrating that aerobic exercise is possible amongst other physical challenges. Neurologically, the post-TBI timeline is complex and may differ by injury severity. TBIs can cause cytotoxic cell death, neuronal damage, neural metabolic crisis, gliotic scarring, inflammation, and an increase in reactive oxygen species, all contributing to adverse outcomes. However, the post-injury timeline is also known to be one of neuroplasticity. It is suggested that neuroplasticity may occur in three phases post-injury (Sophie Su et al., 2016). During the first few days post-injury, increased cell death, neurometabolic strain and decreased cortical inhibitory pathways may signal for neuronal cells and glial cells to replace damaged ones and facilitate recovery, and the utilization of new neuronal networks (Burda and Sofroniew, 2014; Giza and Hovda, 2014; Sophie Su et al., 2016). In the weeks post-injury, synaptogenesis and axonal sprouting assist in the process (Sophie Su et al., 2016), while neural metabolism begins to stabilize (Wieloch and Nikolich, 2006). However, these previous perturbations from the brain’s typical state may induce “negative plasticity,” a perpetuating cycle of reduced brain plasticity, which can persist years after the injury (Figure 3; Tomaszczyk et al., 2014). This negative plasticity has been attributed to lack of use, reduced efficiency in processing sensory and perceptual information, and impaired control over neuromodulation, and is hypothesized to underlay long-term TBI-induced neurodegeneration (Tomaszczyk et al., 2014). Given that aerobic exercise promotes neuroplasticity, it seems especially important to include it in rehabilitation programs at all stages post injury, but it may have the largest benefits at more acute timepoints. It could also be possible that interventions implemented at chronic timepoints should occur at higher frequency, and for longer, to counteract the hypothesized negative plasticity.

Common limitations in this cohort of studies include inadequate comparison groups, lack of details about the exercise programs, small sample sizes, and low adherence rates. While comparison groups are especially important for intervention-based studies, we recognize the many challenges that can occur in this population that can make having these groups challenging (e.g., recruitment windows, scheduling, participants engaging in multiple therapeutic interventions). The importance of adequate comparison groups (e.g., active control, wait-list control) allows us to draw more definitive conclusions about the intervention’s effects, which is especially important post-injury, given that time is an important factor in recovery. Studies which only include one group, or have small sample sizes are still valid and beneficial, and can lead to areas for future research, but they should be interpreted with discretion. Several studies in this review reported adherence rates near 50%, which may indicate challenges with study design, participant interest, feasibility, and accessibility of the intervention. In this set of studies, a mix of clinic-based and home-based studies reported low adherence, therefore, it may be important to offer both in-person and at-home options, such that participants can determine what would be best for them. Given these adherence challenges, it would be worthwhile to conduct feasibility studies in this area to better understand the contributing factors to this low adherence. Another challenge in this set of studies included limited details on aerobic activity, for example, unclear reporting of modality, duration, and frequency of sessions, and if participants had specific effort or heart rate targets. Accurate reporting of interventions allows future researchers to repeat and expand study designs that show promise. While these studies offer valuable insights, the common limitations underscore the necessity for well-structured, detailed research protocols, and robust adherence strategies.

This scoping review serves as a foundation for the application of post-traumatic brain injury aerobic exercise. Based on this review, we propose a methodological framework and a number of suggestions and areas for future research (Table 7). A key finding of this scoping review is the clear lack of research for children aged 12 years and younger, and elderly adults, aged 65 and over. Across all TBI severities, not one study exclusively studied children or elderly adults, despite the fact that these age groups are most at risk for TBIs (Government of Canada, 2020). Interventions at these time points are critical, as children are going through robust neurological development, while older adults typically fare worse after TBI than younger adults (LeBlanc et al., 2006; Araki et al., 2017). TBIs are one of the greatest causes of death and disability in these groups, and adapted aerobic exercise is a promising, and understudied research area. Physical abilities may play a role in this understudied area, but just because someone has reduced or different mobility, does not mean that aerobic exercise should be avoided. Weight-assisted walking, walking poles, water activities and hand cranking are some of the many ways in which researchers could accommodate younger and older age groups into the current body of literature. Another lacking area of research is specific to moderate TBI, or TBIs with a Glasgow Coma Scale rating of 9–13. While individuals with moderate TBIs were included in the research presented, they were either grouped in with severe TBI survivors or their injury severity was unspecified. By definition, moderate TBIs present uniquely upon examination, and survivors deserve tailored and specific research to support their recovery. One recommendation is to include severity-based sub-analyses of research to elicit any severity-specific findings.

**TABLE 7:** Recommendations for future researchers using aerobic exercise as a post-traumatic brain injury intervention.

Item	Recommendation
Study design	• Randomized controlled trial with a minimum of two groups (intervention, control) and two timepoints (baseline, post-intervention)
	• *A priori* power analysis conducted to determine appropriate sample size
Intervention	• Includes a pre-defined aerobic goal (e.g., 80% maximal heart rate)
	• Objective measurement of aerobic goal to assess compliance
	• Any type of aerobic activity
	• At-least 3 × 30 min per week for 12-weeks
	• Supervised activities may increase compliance
Accessibility	• Physical supports and modified activities enhance accessibility
	• Offer in-person and at-home activities
Reproducibility	• Published studies should clearly detail their aerobic methodology such that future studies can build from the work
	• Publish all intervention materials including exercise videos, participant hand-outs to could facilitate future research

## 5 Conclusion

In conclusion, this scoping review presented the array of aerobic interventions used post-TBI across different life stages. It seems that while aerobic exercise post-TBI is a promising intervention area, it is influenced by various factors such as injury severity and the timing of the injury. Moreover, while this review suggests general consensus on the safety and effectiveness of aerobic exercise as a rehabilitative strategy post-TBI, it also highlights some significant gaps and limitations in the current research landscape.

Among these limitations, the inadequacy of comparison groups, small sample sizes, vague reporting on exercise programs, and low adherence rates emerged as key challenges. Despite these hurdles, it’s essential to acknowledge that studies with such limitations can still fuel future research and contribute to our understanding, albeit requiring careful interpretation.

Based on the present review, we suggest several avenues for future research. These include detailed and clear reporting of intervention design and execution, providing both in-clinic and at-home options for participants, and emphasizing the importance of power analysis in determining sample sizes. Furthermore, a focus on age-specific research is paramount given the glaring lack of studies on children aged 12 and under and elderly adults over 65. It is crucial to develop research strategies that are inclusive and accommodate individual abilities across the lifespan, especially considering the high risk of TBIs in these age groups. Also, more specific research on moderate TBIs, often overlooked in studies, is needed to ensure recovery support tailored to this group.

As such, this scoping review serves as a critical cornerstone for future research into the use of aerobic exercise post-TBI. By addressing the noted limitations and gaps in the existing body of knowledge, we can enhance our understanding of this field and optimize recovery strategies for TBI survivors across different ages and injury severities.

## 6 Transparency, rigor, and reproducibility

This review is part of a series of systematic reviews of Brain Changes Initiatives’ Brain Pillars of Health project. This scoping review was conducted according to PRISMA-ScR methodology and guidelines, and our findings are reported according to the PRISMA-ScR checklist. Our review was conducted by a multidisciplinary team including clinical experts in the field of brain injury, researchers and a survivor of brain injury. As reported in this scoping review, a range of aerobic exercise interventions exists for traumatic brain injury rehabilitation, however, there is a paucity of data specifically for children and older adults. Further, we assess intervention methods and study designs currently in use, and suggest standardization practices for future research. The search strategy is detailed in full in this manuscript. Full data charting sheets are available upon request.

## Data availability statement

The original contributions presented in the study are included in the article/Supplementary material, further inquiries can be directed to the corresponding author.

## Author contributions

TS: Conceptualization, Formal analysis, Methodology, Project administration, Visualization, Writing – original draft, Writing – review and editing. JM: Conceptualization, Formal analysis, Investigation, Methodology, Visualization, Writing – original draft, Writing – review and editing. MB: Formal analysis, Investigation, Writing – review and editing. EE: Formal analysis, Investigation, Writing – review and editing. CA: Formal analysis, Investigation, Writing – review and editing. EG: Investigation, Writing – review and editing. HR: Investigation, Writing – review and editing. JB: Investigation, Writing – review and editing. MG: Conceptualization, Funding acquisition, Resources, Writing – review and editing. JG: Conceptualization, Writing – review and editing. BC: Conceptualization, Funding acquisition, Resources, Writing – review and editing.
