# Supplementary material for: Brain changes: aerobic exercise for traumatic brain injury rehabilitation
Source: Front Hum Neurosci. 2023 Dec 20;17:1307507. doi: 10.3389/fnhum.2023.1307507 (PMC10771390; doi:10.3389/fnhum.2023.1307507)
Supplement: Supplementary file 1 [file Table_1.docx]

| *Inclusion Criteria* | *Exclusion Criteria* |
| --- | --- |
| 1. Primary peer reviewed articles | 1. Non-primary research papers |
| 1. English or English-translation available | 1. No English text or translation available |
| 1. Human Participants | 1. Animal studies |
| 1. Published in the year 2000 or onwards | 1. Published before the year 2000 |
| 1. Full text available | 1. No full text available |
| 1. Focus on trauma-induced brain injuries, or trauma-induced brain injured was assessed as a separate group | 1. No separation of other types of brain injuries (*e.g.* stroke, anoxic injuries). Blast injuries were not included in this paper due to their unique mechanism of injury. |
| 1. Focus on aerobic exercise-based interventions following TBI, either using a traditional aerobic activity (*e.g.* walking, jogging, swimming) or the use of heart rate tracking and aerobic heart rate targets | 1. Excludes components or full studies that do not use an aerobic intervention |

Supplementary Table 1. Inclusion and exclusion criteria.
