# Supplementary material for: Brain changes: aerobic exercise for traumatic brain injury rehabilitation
Source: Front Hum Neurosci. 2023 Dec 20;17:1307507. doi: 10.3389/fnhum.2023.1307507 (PMC10771390; doi:10.3389/fnhum.2023.1307507)
Supplement: Supplementary file 2 [file Table_2.docx]

Supplementary Table 2. Search terms and blocks

| *Search Block* | *Modifier* | *Search Terms* |
| --- | --- | --- |
| Traumatic Brain Injury | - | “concuss*” OR “mild traumatic brain injur*” OR “mTBI” OR “head impact” OR “head inj*” OR “brain injuries” OR “brain trauma” OR “head trauma” OR “traumatic brain injury” OR “brain damage” OR “head damage*” OR “cerebr* trauma” OR “craniocerebral trauma” OR “cranial traum*” OR “cranial inj*” |
| Aerobic Exercise | AND | “Aerobic” OR “Aerobic Exercise” OR “Aerobic Intervention” OR “Exercise Intervention” OR “Aerobic Activity” OR “Physical Intervention” OR “Aero*” |
| Intervention | AND | “Recovery” OR “therapy” OR “treatment” OR “Intervention” |
| Review | NOT | “review” OR “meta-analysis” OR “meta review” OR “literature review” |
