# Supplementary material for: Brain changes: aerobic exercise for traumatic brain injury rehabilitation
Source: Front Hum Neurosci. 2023 Dec 20;17:1307507. doi: 10.3389/fnhum.2023.1307507 (PMC10771390; doi:10.3389/fnhum.2023.1307507)
Supplement: Supplementary file 3 [file Table_3.docx]

Supplementary Table 3. Data Charting Items

| *Variable of Interest* | *Details and/or Rationale* |
| --- | --- |
| First Author, Year | This is the first author and year of publication for identification |
| Title | This is the title of the paper for identification |
| Study Design | Type of the study (*e.g.* cohort, RCT) |
| Aim/Objective | Specific study aims related to aerobic exercise |
| Participant demographics | Number of participants; age group and range; sex of participants |
| TBI demographics | Specify if mild, moderate and/or severe was the focus of the study |
| Time since injury | Any pertinent details relating to time since injury |
| Intervention Timeline | Length and number of sessions involved in the intervention |
| Intervention Details | Specific information related to the aerobic component (*e.g.* heart rate goals, exercises done, graded exercises) |
| Intervention Location | Where did participants engage in the intervention? |
| Aerobic modality | Type of exercise |
| Additional Intervention Modalities | Any other activities in the intervention not related to aerobic exercise |
| Participant adherence | How many people followed the intervention as per instructions? |
| General outcome measures | What were the researchers assessing after the intervention? |
| Specific Outcome measures | What specific assessments did the researchers assess? |
| Results Related to Aerobic Exercise | Effects attributed to the aerobic exercise component |
| Considerations | Things needed to know to better interpret the study |
| Notes | Anything else to note about this paper |
